# Flecainide mediated sodium channel blockade enhances blood brain barrier integrity and promotes neuroprotection in neuroinflammation

**DOI:** 10.1038/s41598-025-15430-w

**Published:** 2025-08-23

**Authors:** Mustafa Sindi, Diana Klees, Vera Dobelmann, Paul Disse, Hanne Weigel, Stefanie Lichtenberg, Rebekka Ricci, Leonie Thewes, Gülsüm Deniz-Köseoglu, Christina Hecker, Thomas Müntefering, Andrea Issberner, Joel Gruchot, Hans-Peter Hartung, Tobias Ruck, Carsten Berndt, Thomas Kurz, Holger Stark, Patrick Küry, Britta Engelhardt, Ruth Lyck, Sven G. Meuth, Michael Dietrich, Philipp Albrecht

**Affiliations:** 1 Department of Neurology, Medical Faculty, University Hospital Düsseldorf, Heinrich-Heine-University, Düsseldorf, Germany; 2 Theodor Kocher Institute, University of Bern, Bern, Switzerland; 3 Institute of Pharmaceutical and Medical Chemistry, Faculty of Mathematics and Natural Sciences, Heinrich-Heine University, Düsseldorf, Germany; 4 Department of Neurology, Maria Hilf Clinics, Mönchengladbach, Germany; 5 Core Facility Flow Cytometry, Medical Faculty, University Hospital Düsseldorf, Heinrich Heine University, Düsseldorf, Germany

**Keywords:** Flecainide, Rasagiline, Safinamide, Sodium-channel blocking, MAO-B inhibition, Neuroprotection, Multiple sclerosis, Blood-brain-barrier, EAE, Neuroimmunology, Multiple sclerosis, Blood-brain barrier

## Abstract

Multiple Sclerosis (MS), an autoimmune disorder, is characterized by severe neuroinflammation, leading to demyelination and neuronal damage in the CNS, resulting in significant clinical impairment. MS progression involves complex pathological processes like immune cell invasion and cytokine-mediated recruitment to the CNS. Experimental autoimmune encephalomyelitis (EAE), widely used as a model for MS, despite its translational limitations, has been crucial for identifying effective treatments. Recent studies have shown that sodium channel (NaV) blockers and monoamine oxidase- (MAO) B inhibitors can alleviate symptoms of EAE and optic neuritis (ON), but their mode of action remains partially unclear. To evaluate the effects and understand the action mechanism of NaV blockers and MAO-B inhibitors (rasagiline, safinamide, flecainide and phenytoin) in neurological conditions, various techniques were used, including optical coherence tomography (OCT), optomotor response measurement (OMR), flow cytometry, histological evaluations, Evans blue assay, blood–brain barrier (BBB) permeability assay, western blot, proliferations assay, and gene expression analyses. The study found that the primary therapeutic effect comes from inhibiting the NaV 1.5 sodium channel, not MAO-B inhibition. Flecainide, a NaV 1.5 channel blocker, significantly reduced EAE disability scores, mitigated neurodegeneration, preserved visual function, and restricted immune cell migration into the CNS. Importantly, blocking the NaV 1.5 channel had an effect on the BBB, limiting lymphocyte entry into the CNS. This research highlights sodium channel blockers’ potential in treating EAE. The findings demonstrate induced neuroprotection and reduced disease progression, suggesting a novel therapeutic approach. Crucially, it reveals for the first time that NaV 1.5 channel blockade leads to neuroprotection primarily by affecting the BBB, a key factor in controlling immune cell migration, thus addressing a critical aspect of neuroinflammation.

**Supplementary Information:**

The online version contains supplementary material available at 10.1038/s41598-025-15430-w.

## Introduction

Multiple Sclerosis (MS) is a chronic autoimmune disease that leads to inflammation, demyelination, loss of oligodendrocytes, axonal injury, and progressive neuronal degeneration within the central nervous system (CNS)^1^. The degenerative processes contribute significantly to the ongoing disability seen in MS patients, which often proves resistant to typical immunosuppressive and immunomodulatory treatments. Pathological changes in MS are commonly associated with lymphocyte and macrophage infiltration into CNS tissue, driving the expression of adhesion molecules and encouraging immune cell migration through the release of inflammatory cytokines. This chain of events triggers damage to glial cells and neurons, although the exact mechanisms remain incompletely understood. It is suggested that oxidative stress plays a crucial role in neuronal injury, as immune cells activated in this process may release reactive oxygen and nitrogen species^2^.

Additionally, the MultipleMS Consortium^3^ highlighted that the varying severity of MS among patients could be due to distinct genetic variants, which act primarily independent of immune responses, suggesting that CNS resilience plays a crucial role in the progression of MS. In light of these findings, MS’s intricate nature, influenced by genetic, environmental, and lifestyle factors, necessitates ongoing research for accurate diagnosis and effective treatment, with a primary aim to improve the quality of life for patients battling this disease.

Recent studies have highlighted additional mechanisms contributing to neuronal injury and disease progression in MS. Acid-sensing ion channels, particularly ASIC1, have been implicated in axonal degeneration through proton-gated sodium and calcium influx during tissue acidosis, as shown in experimental autoimmune encephalomyelitis (EAE), an animal model for MS. The disruption of ASIC1 or its pharmacological inhibition using amiloride conferred neuroprotection without altering CNS inflammation, suggesting a potential therapeutic avenue for MS^4^. Similarly, the transient receptor potential vanilloid-type 4 (TRPV4) ion channel has been identified as a mediator of blood–brain barrier (BBB) dysfunction. Endothelial TRPV4 expression, upregulated in response to microglia-derived TNFα, exacerbates T cell extravasation and neuroinflammation, emphasizing its role in MS pathogenesis^5^. Furthermore, Th17 cells have been found to release glutamate via β1-integrin-and KV1.3 channel-dependent signaling, inducing calcium-mediated neuronal damage. Inhibition of KV1.3 or glutaminase has shown promise in reducing glutamate-mediated toxicity and improving outcomes in EAE^6^. Finally blocking potassium channels with 4-aminopyridine has demonstrated protective effects in EAE and preclinical optic nerve damage^7^. These findings underscore the multifaceted roles of ion channels and immune cell signaling in driving neuroinflammation and neurodegeneration.

Sodium channel blockers are distinguished by their varied pharmacological properties, primarily modulating membrane action potentials through different mechanisms. These agents are categorized into subclasses based on their specific effects, with some exhibiting dual inhibitory actions, such as targeting sodium channels and inhibiting monoamine oxidase (MAO)-B activity. Others demonstrate selective affinity, primarily inhibiting sodium channels. The specificity among these blockers ranges from compounds targeting particular sodium channel subtypes to those affecting multiple types indiscriminately. Recent research has sparked interest in these blockers due to preliminary evidence of their beneficial effects in EAE and optic neuritis (ON), though their underlying mechanisms remain largely unexplored.

The intricate nature of EAE has prompted a myriad of investigations into its etiology and treatment. The selective MAO inhibitor, rasagiline, has been shown to induce neuroprotection in animal models of Parkinson´s disease^8,9^, indicative of the potential therapeutic efficacy of MAO inhibitors. NaV blockade, exemplified by non-selective NaV-blocker phenytoin, has also been proposed as a potential approach for MS treatment, as it protects axons from degeneration by averting an energy deficit^10^ and suppresses the activation of innate immune cells in the CNS^11^. In addition, research points to direct effects on microglial cells^12^ and a reduction in CD45+infiltrates in the CNS upon phenytoin treatment. Safinamide, a combined MAO-B inhibitor and non-selective sodium channel blocker, has also shown promising results, as it diminished the formation of superoxide and increased GSH-level in microglia culture, leading to reduced CD68+monocytes in the spinal cord of EAE mice^13^. Similarly, flecainide, a sodium channel blocker, has also been found to reduce CD68+cell activation/infiltration^13^.

Beginning with evaluating their overall therapeutic benefits, we will progress to a detailed analysis of the most effective compound, focusing on its mechanism of action in an inflammatory setting. This understanding is crucial for exploring their potential in treating neuroinflammatory diseases. The findings from this research could significantly advance MS treatment strategies and contribute to the field.

## Material and methods

### Animals, animal models, treatment

Female, six-week-old C57BL/6J mice were purchased from Janvier Labs (Le Genest-Saint-Isle, France). The NOD/ShiLtJ mice (Female, six-week-old) originate from the internal breeding facility (ZETT) at the University of Düsseldorf. EAE mouse model.

Mice were immunized with 200 μg of myelin oligodendrocyte glycoprotein fragment 35–55 (MOG35–55), purchased from BIOTREND emulsified in 200 μl of complete Freund’s adjuvant (CFA), supplemented with 800 μg of heat-killed Mycobacterium tuberculosis (MT) H37Ra, both purchased from BD Difco (injected subcutaneous, distributed over four spots on the hind and front flank) and additional intraperitoneal injections of 200 ng of pertussis toxin (PTX) from Sigma-Aldrich on days 0 and 2 post immunization (p.i.). The sham control group (sham) also received PTX and CFA, but no MOG35–55 peptide. The substances used for treatment, their concentration, mode of action, treatment interval and treatment start are described in Table 1. The used concentrations for each substance were adapted from recent dose finding studies found in the literature investigating the optimal dose.

**Table 1:** Substance treatment details.

Compound	Rasagiline	Safinamide	Flecainide	Phenytoin
Administration form	Oral administration	Oral administration	Subcutaneous injection	Subcutaneous injection
Concentration	5 mg/kg/bw	8 mg/kg/bw	30 mg/kg bw	50 mg/kg bw
Mode of action	Selective MAO-B inhibition	Reversible MAO-B inhibition Sodium and calcium channel blocking Glutamate release inhibition Dopamine and serotonin reuptake inhibition	Selective NaV1.5 sodium channel blocker	Unselective NaV1.1, NaV1.2, NaV1.3, NaV1.5 and NaV1.6 sodium channel blocker
Treatment interval	Daily	Daily	Daily	3 times/week
Treatment start	Directly p.i	Directly p.i	Directly p.i	Directly p.i
CAS Corresponding treatment reference	161735-79-1 (Sigma Aldrich) ^14,15^	202825-46-5 (Sigma Aldrich) ^16^	54143-56-5 (Sigma Aldrich) ^13^	630-93-3 (Sigma Aldrich) ^11^

The experimental protocol was reviewed and approved by the “State Office for Nature, Environment and Consumer Protection of North Rhine-Westphalia, Germany “Landesamt für Natur, Umwelt und Verbraucherschutz Nordrhein-Westfalen (LANUV) under approval number Az. 81-02.04.2019.A063.

The in vitro concentrations of 2 µM and 5 µM flecainide used for treating primary mouse brain microvascular endothelial cells (pMBMECs) were selected as part of a dose-finding approach based on previously published studies^17,18^. These concentrations were chosen to approximate pharmacologically relevant levels corresponding to the systemic in vivo dosage of 30 mg/kg administered subcutaneously in our EAE model, while allowing the assessment of dose-dependent effects on endothelial gene expression and barrier function.

### OCT measurements

Periodic OCT measurements were conducted using the Spectralis^®^ HRA+OCT device (Heidelberg Engineering, Germany) with several adaptations for rodents, as previously described^19^. Segmentation of retinal volume scans was performed using the Heidelberg Eye Explorer software, with manual control for segmentation errors. The volume of the parapapillary region was assessed using the ETDRS grid, excluding the center with the disc, as published previously^20^. Inner retinal layer (IRL) thickness was examined at defined intervals after irradiation and compared with baseline measurements (IRL: NFL, GCL, and IPL layer).

For the measurements, the animals were anesthetized using isoflurane (Vaporizer from Harvard Apparatus Anesthetic Vaporizors; Isofluran from Piramal critical care). Specifically, induction was carried out at 3.5% isoflurane, followed by maintenance at 2% isoflurane, with a flow rate of 0.6 L/minute of oxygen. The gas was transferred through nose cones to the mice.

### Optomotor response (OMR) measurements

OMR measurements were performed periodically, exclusively in C57BL/6J mice, using the OptoMotry^®^ device from Cerebral Mechanics, in parallel with OCT measurements. Spatial frequency was monitored as a parameter for visual function. The spatial frequency threshold was determined by randomly changing the spatial frequency to identify the threshold at which the mouse could track the grids, as previously described^20,21^.

### Histology (optic nerves, retinal cross sections and retinal whole mounts)

Mice were euthanized using 100 mg/kg Ketamine und 20 mg Xylazin intraperitoneal (i.p). (in 250 µl NaCl 0,9%) followed by cardiac perfusion using phosphate-buffered saline (Gibco, Carlsbad, USA).

The optic nerves were then isolated and fixated in 4% paraformaldehyde (Carl Roth, Karlsruhe, Germany) overnight. After fixation, the optic nerves were subjected to a sucrose gradient for dehydration and subsequently embedded in O.C.T. compound (Sakura™ Finetek, Alphen aan den Rijn, Netherlands). Longitudinal sections of five micrometers were cut and prepared for fluorescence staining. Longitudinal sections of the optic nerves were used for quantifying T-Lymphocytes (CD3 (Clone 17A2), 1:400, Biolegend), assessing the state of myelination (MBP (Clone 12), Merck Millipore, 1:500), evaluating microglial activation (Iba1 (Clone GT10312), 1:500, Merck) using Leica HyD detector attached to a Leica DMi8 confocal microscope (63 × objective lens magnification). Astrocytic activation (GFAP (Clone 173,004), 1:1000 Synaptic System) was assessed using retinal cross sections. Cy3 goat anti-mouse (1:500 Millipore), Cy3 goat anti-rat (1:500, Millipore) and Cy3 goat anti-rabbit (1:500, Invitrogen) were used as secondary antibodies. The numbers of cells stained with CD3 and Iba1 were analyzed using ImageJ software, applied by blinded raters, and expressed as a ratio to DAPI staining. The overall signal for MBP (positive total area in the red channel) was analyzed using ImageJ software. Cell counts for CD3 and Iba1, as well as the positive area for MBP and GFAP, were assessed in the whole field across 6–9 images per sample. The mean values were calculated and utilized for analysis.

RGC count was calculated by a semi-automated count of Brn3a+cells on retinal flat mounts. Briefly, retinae were stained with Brn3a (1:200, Santa Cruz Biotechnology, cat# sc-31984) antibody and flat-mounted on glass slides. Each retina was then divided into four quadrants (three areas per quadrant: central, mid-periphery, and far-periphery). For each eye, Brn3a+cell count was summed up from all 6–12 areas imaged.

### Evan’s Blue Dye assay

Evan’s Blue Dye (EBD) is a diazo dye that binds to serum albumin, creating a large molecular complex that normally does not cross the intact BBB. However, in pathological conditions leading to increased BBB permeability, the EBD-albumin complex can cross the BBB and accumulate in brain tissue, thus providing a measurable indication of barrier disruption. At the pre-determined time points of 18 days post-immunization, the mice were injected intravenously with 4% Evan’s Blue Dye in saline at a dosage of 4 ml/kg body weight. The dye was allowed to circulate for 3 h to ensure systemic distribution. After the circulation period, the mice were anesthetized and transcardially perfused with phosphate-buffered saline (PBS) to remove intravascular dye. The brains and spinal cords were then carefully extracted, weighed, and homogenized in N,N-dimethylformamide to extract the dye from the tissue. The samples were then centrifuged, and the supernatants were collected for spectrophotometric analysis. Quantification of the EBD was performed using a TECAN spectroscopic device. The absorption of the extracted solutions was measured at 565 nm, a wavelength at which EBD exhibits a distinct peak. EBD concentration was calculated from the absorbance values using a standard curve generated with known concentrations of EBD. The amount of EBD in the brain tissue was then expressed as µg of EBD per g of brain tissue. All data were collected and analyzed using appropriate statistical methods.

### Flow cytometry analysis

Flow cytometry was used to examine the lymphocyte subpopulations in spinal cord and spleen of the EAE mice at specified time points. Spinal cords were carefully harvested and mechanically and enzymatically (Collagenase, DNAse) dissociated. Lymphocytes were then isolated from the suspension using a LymphoprepTM-gradient (Stemcell technologies). Spleen was collected and mechanically dissociated by passing it through a 70 µm cell strainer. Red blood cells were lysed with ACK Lysis Buffer and suspension was again passed through a 70 µm cell strainer.

Lymphocytes from both organs were centrifuged and resuspended in FACS buffer, containing 2 mM EDTA and 2% fetal calf serum. Cells were then stained with fluorochrome-conjugated monoclonal antibodies (Tables 2, and 3, respectively) in the dark 4 °C for 30 min. The cells were then washed with FACS buffer and prepared for analysis. Cells were analyzed on a CytoFLEX S (Beckman Coulter) and data was interpreted using Kaluza Analysis Software (Beckman Coulter). Lymphocytes were identified based on forward and side scatter properties, with specific populations determined by surface marker expression (representative gating strategy is stated in Fig. S1). Data are reported as the total cell counts (via flowrate check) of each cell type within the total lymphocyte population.

**Table 2:** Antibodies used for flow cytometry analysis on CNS immune cell infiltration analysis (ex vivo experiment).

Fluorochrome	Antigen	Clone	Company
BrilliantViolet421	CD4	RM4-5	BD Biosciences
BrilliantViolet605	CD8	53-6.7	Biolegend
BrilliantViolet650	CD3	17A2	Biolegend
FITC	CD11b	M1/70	Biolegend
PE-Cy5.5	Ly6G	1A8	Biolegend
PE	CD11c	N418	Biolegend
ECD	CD19	6D5	Biolegend
PE Dazzle594	CD25	PC61	BD Biosciences
APC	NK1.1	S17016D	Biolegend
Alexa Fluor 700	Ly6C	HK1.4	Biolegend
APC-Cy7	CD45	30-F11	Biolegend

**Table 3:** Antibodies used for flow cytometry analysis on lymphocyte activation, adhesion, and proliferation (in vitro experiment).

Fluorochrome	Antigen	Clone	Company
eFluor450	CD69	H1.2F3	Invitrogen
Fluor506	Viability dye	–	Invitrogen
BV605	CD8a	53-6.7	BioLegend
FITC	CD19	MB19-1	BioLegend
PerCP-Cy5.5	CD3	17A2	BioLegend
PE	CD11a	2D7	BD Biosciences
PE-Cy7	CD25	PC61	BioLegend
Alexa Fluor 647	CD4	RM4-5	BioLegend
APC/Fire750	CD49d	R1-2	BioLegend
PE-Dazzle594	Ki-67	16A8	BioLegend

### Isolation of pMBMECs

pMBMECs were isolated according to different protocols for performing the quantitative PCR or the permeability assay. In both procedures, the pMBMECs were never passaged between isolation and experiment. Flecainide treatment was applied for 24 h prior to qPCR analyses and for 7 days prior to Western blot analyses, starting after pMBMECs had reached confluency.

#### Isolation pMBMECs for quantitative PCR and western blot

We began by euthanizing six- to eight-week-old female C57BL/6 mice. After euthanasia, the brains were carefully extracted and immediately transferred onto sterile filter paper to facilitate the removal of the meninges. Following this step, the brain tissue was homogenized to a uniform consistency and subsequently processed according to the protocol of the Adult Brain Dissociation Kit (Miltenyi Biotec), which is optimized for effective dissociation of murine brain tissue. To isolate microvascular endothelial cells, the resulting single-cell suspension was subjected to magnetic-activated cell sorting (MACS). First, CD45^+^ immune cells were labeled using CD45 MicroBeads and removed by magnetic separation via LS columns and the MACS Separator. The flow-through, containing CD45^−^ cells, was then incubated with CD31 MicroBeads to isolate CD31^+^ endothelial cells. These CD45^−^CD31^+^ cells were collected and plated in 12-well plates for culture. Prior to seeding, the plates were coated overnight at 4 °C with a coating solution consisting of 500 µl dH_2_O, 400 µl collagen, and 100 µl fibronectin (200 µl per well), to enhance cell adhesion. The isolated endothelial cells were seeded in 1.5 ml of MBMEC medium per well. For the initial two days, cells were cultured in MBMEC medium supplemented with 0.1% pyromycin (10 µl pyromycin per 10 ml medium) to select for the desired cell population. Thereafter, the medium was replaced with pyromycin-free MBMEC medium. The MBMEC culture medium consisted of 40 ml DMEM high glucose, 10 ml fetal calf serum (FCS), 25 µl basic fibroblast growth factor (bFGF) and 50 µl heparin.

#### Isolation of pMBMECs for permeability assay

Cells were isolated by the method of Coisne et al.^22^ as follows: for each preparation cortices from six- to ten-weeks old gender matched mice were isolated and meninges were removed. Preparations were pooled and homogenized in Hank’s balanced salt solution containing 0.1% bovine serum albumin. The homogenate was mixed with 30% dextran and centrifuged at 3000g for 25 min at 10 °C. The pellet containing the vascular fraction was collected. Centrifugation and pellet harvesting was repeated once. The collected vascular fraction was then filtered through a 60 μm nylon mesh. The capillary-enriched filtrate was digested in DNase I (10 mg/mL), TLCK (0.147 mg/mL), and collagenase/dispase (2 mg/mL) for 30 min at 37 °C. The digestion was stopped by an excess of wash buffer and filtered through a 20 μm nylon mesh. The crude cell preparation of pMBMECs were cultured for 48 h in the presence of 4 µg/ml puromycin, which allowed selective growth of pMBMECs only.

### Western blot analysis of protein expression in pMBMECs

pMBMECs were cultured to confluence as described in “Isolation pMBMECs for quantitative PCR and western blot” section, then treated for 7 days with flecainide (2 µM or 5 µM) or vehicle. After treatment, cells were washed with PBS and lysed in NP-40 buffer (150 mM NaCl, 50 mM Tris/HCl, 1% NP-40, pH 8.0) on ice for 20 min and then scraped off using a pipette tip. Lysates were cleared by centrifugation (12,000 × g, 10 min, 4 °C), and protein concentration was determined using the BCassay kit (Interchim, France). Equal protein amounts (25 µg) were mixed with 1 × Laemmli buffer, denatured at 95 °C for 5 min, separated by SDS-PAGE, and transferred onto 0.2 µm nitrocellulose membranes. Membranes were blocked for 10 min at room temperature with EveryBlot buffer (Bio-Rad, USA), then incubated overnight at 4 °C with the following primary antibodies: anti-Actin (Invitrogen, PA1-183, 1:4000), anti-PECAM1 (Antibodies Online, ABIN669006, 1:1000), anti-β-Integrin (ABIN739029, 1:1000), anti-JAM3 (ABIN1386406, 1:1000), and anti-JAM2 (ABIN3187667, 1:1000). Following three washes with PBS containing 0.05% Tween-20, membranes were incubated for 1.5 h on a shaker at room temperature with IRDye-labeled secondary antibodies: Goat anti-Rabbit 680RD (LI-COR, 926-68071) and Goat anti-Mouse 800CW (LI-COR, 926-32210). Protein bands were visualized using the ChemiDoc system (Bio-Rad) and quantified with ImageLab software. Expression levels were normalized to β-actin and analyzed using GraphPad Prism.

### Quantitative PCR analysis

For our qPCR analysis, we utilized the QuantStudio 3 from Thermo Fisher Scientific, employing SYBR Green as our detection chemistry. After preparing and loading our samples, we conducted the qPCR run analyzing the genes listed in Table 4 (in different experimental approaches). The machine’s sophisticated technology measured DNA quantity using the fluorescence of the SYBR Green. Post-run, we processed and interpreted the data using the QuantStudio Design and Analysis Software from Thermo Fisher Scientific, enabling us to calculate relative gene expression levels using the ΔΔCt and ΔCt, respectively.

**Table 4:** List of primers used for qPCR analysis.

V-CAM-1	JAM-1
Forward sequence GCTATGAGGATGGAAGACTCTGG	Forward sequence CACCTACTCTGGCTTCTCCTCT
Reverse sequence ACTTGTGCAGCCACCTGAGATC	Reverse SEQUENCE TGCCACTGGATGAGAAGGTGAC
I-CAM-1	JAM-2
Forward sequence AAACCAGACCCTGGAACTGCAC	Forward sequence CAGACTGGAGTGGAAGAAGGTG
Reverse sequence GCCTGGCATTTCAGAGTCTGCT	Reverse sequence GCTGACTTCACAGCGATACTCTC
I-CAM-2	JAM-3
Forward sequence GACGGTCTCAACTTTTCCTGCC	Forward sequence GCATTGCTTCCAATGACGCAGG
Reverse sequence CCATTTGGTTGTCCTGCATCGG	Reverse sequence GATGAAGCAGCCTCGTCTGTAC
PECAM-1	Occludin
Forward sequence CCAAAGCCAGTAGCATCATGGTC	Forward sequence TGGCAAGCGATCATACCCAGAG
Reverse sequence GGATGGTGAAGTTGGCTACAGG	Reverse sequence CTGCCTGAAGTCATCCACACTC
NaV1.5	NaV1.9
Forward sequence GGTTCCGAGATGGTCAGTTGCT	Forward sequence GTCTTCAGCATGTTCATTATC
Reverse sequence CAAGGACTCCTGTCCAATACGG	Reverse sequence CCACACATTATGAAGTCCTCG
NaV1.6	GAPDH
Forward sequence AAGTGGACAGCCTATGGCTTC	Forward sequence TGTGTCCGTCGTGGATCTGA
Reverse sequence AGCCAGAAGATGAGACACACC	Reverse sequence CCTGCTTCACCACCTTCTTGA

The treatment groups in the in vitro treatment experiments included cells exposed to 2 µM or 5 µM flecainide or PBS vehicle control 24 h prior to cell harvesting. The in vitro concentrations were used based on previous experiments with flecainide found in literature^17,18^.

### Permeability assay

Permeability assays were performed in triplicates as reported by Coisne et al.^22^, with minor adaptations: pMBMECs were grown on Matrigel-coated Transwell^®^ filter inserts (0.4 μm pore size, 6.5 mm diameter; article number 662640, Greiner Bio-One Vacuette Schweiz GmbH, St. Gallen, Switzerland) for 6 to 8 days. Alexa Fluor 680-dextran (3 kDa, 10 μg/ml; LuBioScience, Luzerne, Switzerland) was used as permeability tracer. Diffused dextran was quantified using the Odyssey Imaging System (LI-COR, Bad Homburg, Germany) and the clearance value (*Pe*, in cm/min) of the pMBMECs calculated as reported by Coisne et al. 2005. Flecainide treatment at 5 µM was for 24 h and IL-1β treatment at 20 ng/ml was for 16 h. The in vitro concentrations of flecainide were used based on previous experiments found in literature^17,18^. After the experiment, each filter was examined for confluent growth of pMBMECs by staining with phalloidin-rhodamine and subsequent fluorescence microscopy. The inflammatory state of pMBMECs after IL-1β stimulation was monitored in parallel samples by staining with the homemade rat anti-mouse ICAM-1 monoclonal antibody 29G1 followed by a secondary donkey anti-rat Cy5 antibody (Jackson ImmunoResearch, Milan Analytica AG, Rheinfelden, Switzerland).

### Isolation of splenocytes for proliferation assay and qPCR analysis

Immediately following euthanasia, spleens were aseptically removed and placed into wash buffer composed of DMEM supplemented with fetal calf serum and antibiotics. For tissue dissociation, each spleen was transferred onto a pre-wetted 40 µm cell strainer positioned on a conical tube. The spleen was mechanically dissociated by gently pressing it through the strainer using the plunger of a syringe. The cell strainer was then rinsed with additional wash buffer to collect all cells. The resulting single-cell suspension was centrifuged at 4 °C. After removal of the supernatant, the cell pellet was resuspended in pre-warmed ACK lysis buffer to lyse erythrocytes. The suspension was incubated at room temperature, and erythrocyte lysis was subsequently stopped by adding wash buffer, followed by a second centrifugation step at 4 °C. The final cell pellet was resuspended in wash buffer and passed through a freshly rinsed 40 µm cell strainer into a new conical tube to ensure maximal recovery of splenocytes.

### Proliferation assay using CFSE and Ki-67

To investigate the effects of flecainide on lymphocyte proliferation, we performed a combined assay using CFSE (carboxyfluorescein succinimidyl ester) labeling and intracellular Ki-67 staining. Splenocytes were isolated as described in “Isolation of splenocytes for proliferation assay and qPCR analysis” section. Prior to culture, cells were labeled with CFSE by incubation in PBS supplemented with fetal calf serum and the dye at 37 °C in the dark. The labeling reaction was quenched by the addition of cold wash medium, followed by incubation on ice. After two washing steps, cells were resuspended in murine T cell medium composed of IMDM supplemented with fetal calf serum, β-mercaptoethanol, L-glutamine, and antibiotics. T cell activation was achieved by seeding the CFSE-labeled splenocytes into anti-CD3-coated 96-well plates. Cells were treated with either vehicle (containing 0,005% DMSO), 2 µM flecainide, or 5 µM flecainide and incubated for five days at 37 °C in a humidified CO_2_ incubator. On day three, half of the culture medium was carefully replaced with fresh treatment medium. At the end of the incubation period, proliferation was assessed by flow cytometric analysis of CFSE dilution and intracellular Ki-67 expression. For Ki-67 staining, cells were fixed, permeabilized, and stained with a fluorochrome-conjugated anti-Ki-67 antibody according to the manufacturer’s protocol. Data were acquired and analyzed within defined immune cell subsets.

### Statistics and data interpretation

Statistical analysis was performed using Prism (version 9, Graphpad Software, Inc.) and IBM SPSS Statistics (version 20, IBM Corporation, USA). Total and percent changes of the acquired retinal parameters (OCT and OMR) were analyzed using generalized estimation equation (GEE) models, accounting for within-subject inter-eye correlations, to test for differences between the two groups. For non-paired data, group means analyses were compared using a one-way ANOVA with the Dunnett´s post hoc test, utilizing one optic nerve per animal for the histological investigations. The thickness of the inner retinal layers, ranging from the inner limiting membrane to the bottom of the inner plexiform layer, the total retinal thickness and outer retinal layers were assessed in volume scans around the optic disc as the primary OCT-based outcome parameters. The spatial frequency served as a functional primary readout. The other histology-derived parameters were assessed as secondary outcome criteria. The severity of EAE symptoms, extending from minor hind limb weakness to complete paralysis, was assessed through a standardized scoring system (ranging from 0 to 5), providing a quantitative outcome measure of neuroinflammation and neurodegeneration. The EAE scores served as an integral part of the study, illustrating the clinical manifestation and progression of the disease, offering a real-time evaluation of the neurological impairment. QPCR was deployed for the precise quantification of targeted gene expression changes. The relative changes in gene expression levels were examined as one of the primary outcome measures, offering key insights into the molecular mechanisms. The permeability of the BBB was further evaluated by using the Evans Blue assay. This approach involved the systemic administration of Evans Blue dye, the penetration of which into the brain tissue served as a direct indicator of BBB disruption. The quantified extent of dye penetration, extracted and measured spectrophotometrically, offered a primary outcome measure of BBB integrity. The data derived from the Evans Blue assay yielded essential insights into the degree and timing of BBB permeability changes in response to neuroinflammation and following various therapeutic interventions.

## Results

### Assessment of clinical disability (EAE-scoring)

We aimed at determining the most promising pure sodium channel blocker for extended EAE studies in various rodent strains by conducting a preliminary screening experiment to discern the more effective candidate between flecainide and phenytoin. Both these drugs have been previously noted for their beneficial effects in EAE models, with flecainide^10,13^ being a selective sodium channel blocker and phenytoin^11,23^ an unselective one.

**Fig. 1:**
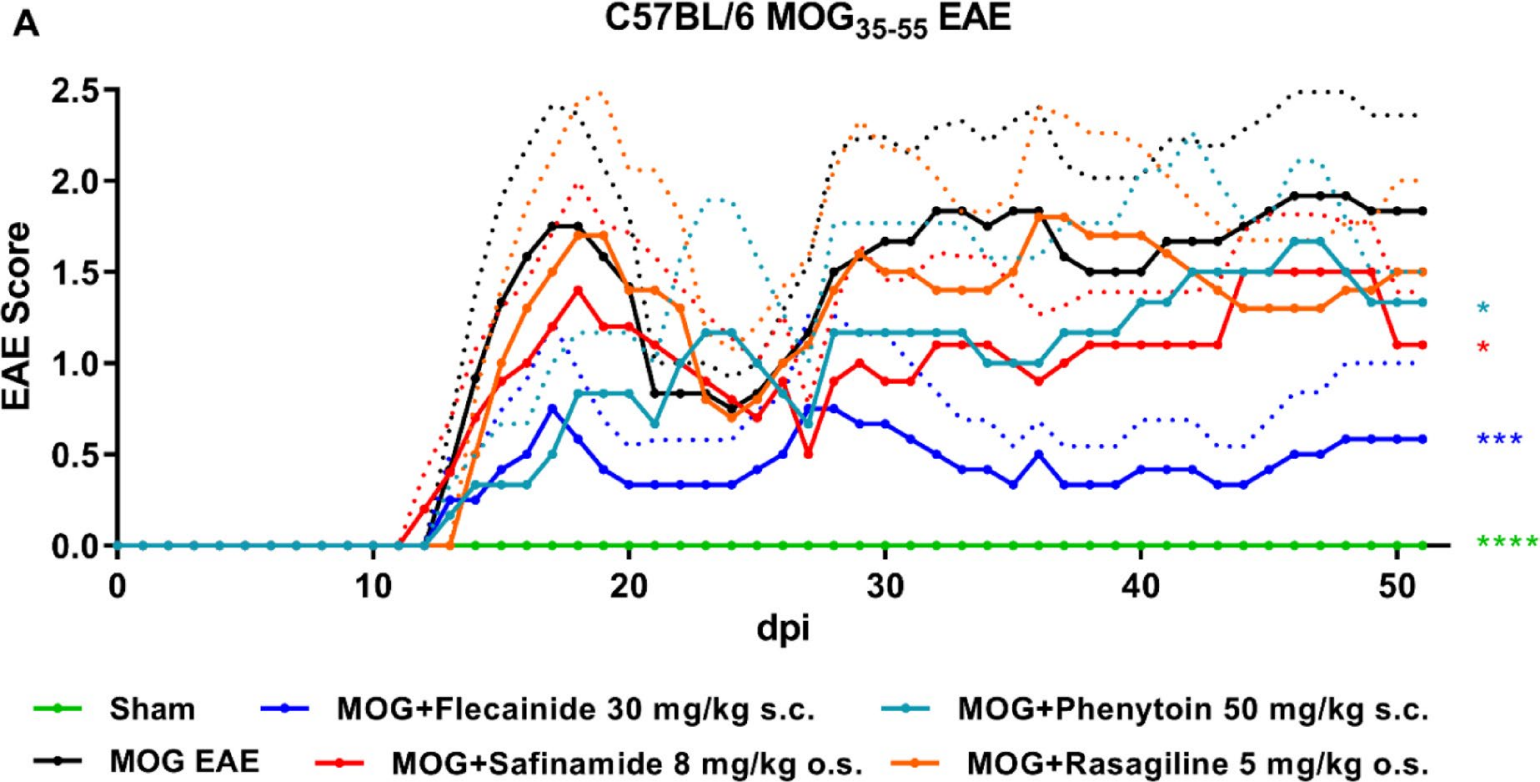
Flecainide shows superior beneficial effects in C57BL/6J EAE compared to phenytoin. Progression of EAE in C57BL/6J mice over 52 days post EAE induction, immunized with MOG35-55. Treatment with flecainide, safinamide, phenytoin and rasagiline was initiated from day 0. *p < 0.05; ***p < 0.001, ****p < 0.0001, area under the curve analyzed via ANOVA with Dunnett’s post hoc test in comparison to MOG EAE. n = 6 per group. Dotted lines represent the standard deviation.

Figure 1 demonstrates our experiment comparing two drugs’ effectiveness in alleviating EAE symptoms, guiding future long-term study decisions. Flecainide emerged as the more effective of the two, demonstrating a superior ability to alleviate EAE conditions. This was quantitatively reflected in the mean difference (MD) of 0.4551 (± 0.0854, *P*: 0.0003), favoring flecainide over phenytoin. This led us to our decision to prioritize it in subsequent, more comprehensive EAE studies across different rodent strains. Rasagiline demonstrated no positive effects, whereas safinamide slightly improved EAE outcomes compared to the MOG vehicle (MD: 0.3196 ± 0.1249, *P*: 0.0120).

C57BL/6J and non-obese-diabetic (NOD) mice exhibit distinct EAE progression patterns: the former develops a more chronic progression with a disease peak around day 18 post-immunization, while the latter shows a relapsing–remitting course with complete recovery phases. In the mentioned subsequent studies, the impact of flecainide, rasagiline, and safinamide on EAE progression in these mouse models was evaluated over a period of 90 days p.i. of EAE, induced by immunization with MOG35-55, scored clinically over time. The treatment groups were compared to the MOG EAE vehicle group, with n = 9 per group. The treatments were initiated from day 0 of immunization.

**Fig. 2:**
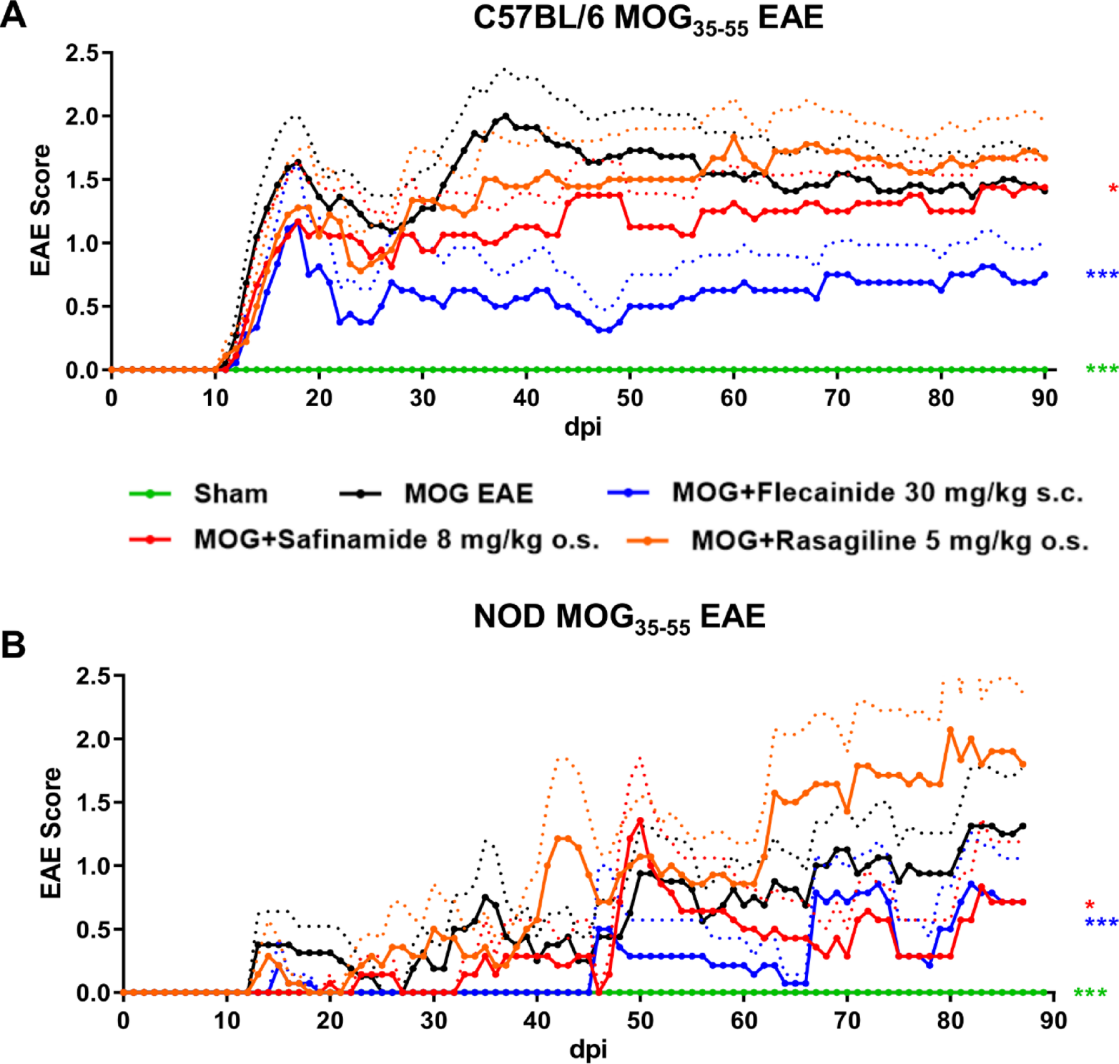
Flecainide and safinamide mitigates clinical long-term EAE severity. Progression of EAE in C57BL/6J mice (**A**) and NOD female mice (**B**) over 90 days post EAE induction, immunized with MOG35-55. Treatment with flecainide, safinamide, and rasagiline was initiated from day 0. **p < 0.01; ***p < 0.001, area under the curve analyzed via ANOVA with Dunnett’s post hoc test in comparison to MOG EAE. n = 9 per group. Dotted lines represent the standard deviation.

Figure 2A illustrates the clinical scores of EAE in the acute progressive C57BL/6J mouse model. Administration of flecainide (30 mg/kg subcutaneously) and safinamide (8 mg/kg orally) resulted in a significant reduction in EAE-related disability. The flecainide-treated group exhibited a MD score of 0.7711 ± 0.0649 (*P* = 0.0002), while the safinamide-treated group showed an MD score of 0.2930 ± 0.0753 (*P* = 0.0251). These findings indicate a substantial decrease in disability with both flecainide and safinamide treatment compared to the MOG-induced EAE control group.

Similarly, Fig. 2B presents the EAE clinical scores in the chronic progressive NOD mouse model. Notably, treatment with flecainide (MD: 0.3335 ± 0.0528, *P* = 0.0010) and safinamide (MD: 0.2437 ± 0.0545, *P* = 0.0126) also significantly reduced EAE-related disability compared to the MOG EAE control group.

In contrast, treatment with rasagiline did not result in a significant reduction in disability in either mouse model compared to the untreated MOG EAE group.

### Evaluation of neurodegeneration and visual function via OCT and OMR

**Fig. 3:**
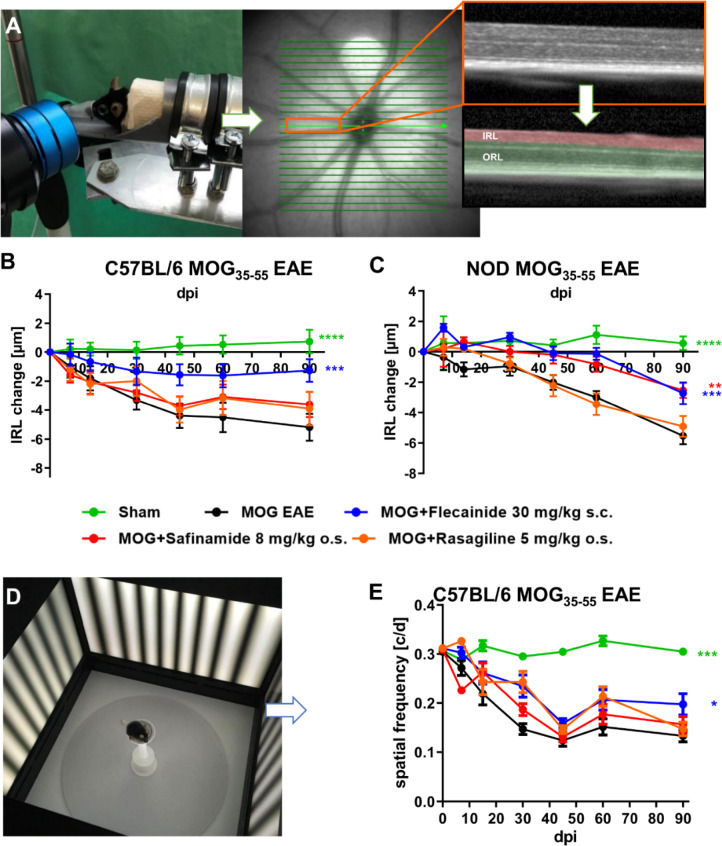
Longitudinal evaluation of neurodegeneration and visual function via OCT and OMR reveals beneficial effects of flecainide and safinamide. OCT volume scan (**A**) of the degeneration of the inner retinal layers (IRL) (**B**, **C**), and OMR measurement of visual function (spatial frequency) (**D**) in cycles per degree (c/d) (**E**) in female C57BL/6J-EAE and NOD EAE mice over 90 days post EAE induction. **p < 0.01; ***p < 0.001, ****p < 0.0001, area under the curve analyzed via ANOVA with Dunnett’s post hoc test in comparison to MOG EAE. n = 9 per group.

In addition to assessing the effects of flecainide and safinamide on EAE-related disability, we conducted a longitudinal evaluation of neurodegeneration and visual function using OCT in both C57BL/6J and NOD EAE mice, as well as OMR testing in C57BL/6J EAE mice over 90 days post EAE induction (Fig. 3). NOD mice were not subjected to OMR testing, as their genetic background is associated with congenital visual impairment, limiting the reliability of behavioral vision assessments. The OCT volume scans examined the degeneration of the inner retinal layers (IRL). In C57BL/6J EAE mice, flecainide treatment significantly reduced the change in IRL thickness over the 90-day period (MD = 1.940 ± 0.7879, *P* = 0.0004), whereas safinamide and rasagiline showed no significant differences compared to the MOG EAE vehicle group. Similarly, in NOD EAE mice, flecainide (MD = 1.844 ± 0.8840, *P* = 0.0162) and safinamide (MD = 1.473 ± 0.8223, *P* = 0.0061) both significantly reduced IRL thickness changes. Flecainide treatment led to a significant improvement in visual function compared to the MOG vehicle group (MD = 0.0458 ± 0.0347, *P* = 0.0111), while safinamide and rasagiline did not show significant effects. These findings indicate that flecainide effectively slows neurodegeneration and preserves visual function in EAE mouse models, and safinamide also provides significant neuroprotective effects in NOD EAE mice, supporting their potential therapeutic benefits in neurodegenerative conditions like EAE.

### Histological analyses of immunological markers in the optic nerve

**Fig. 4:**
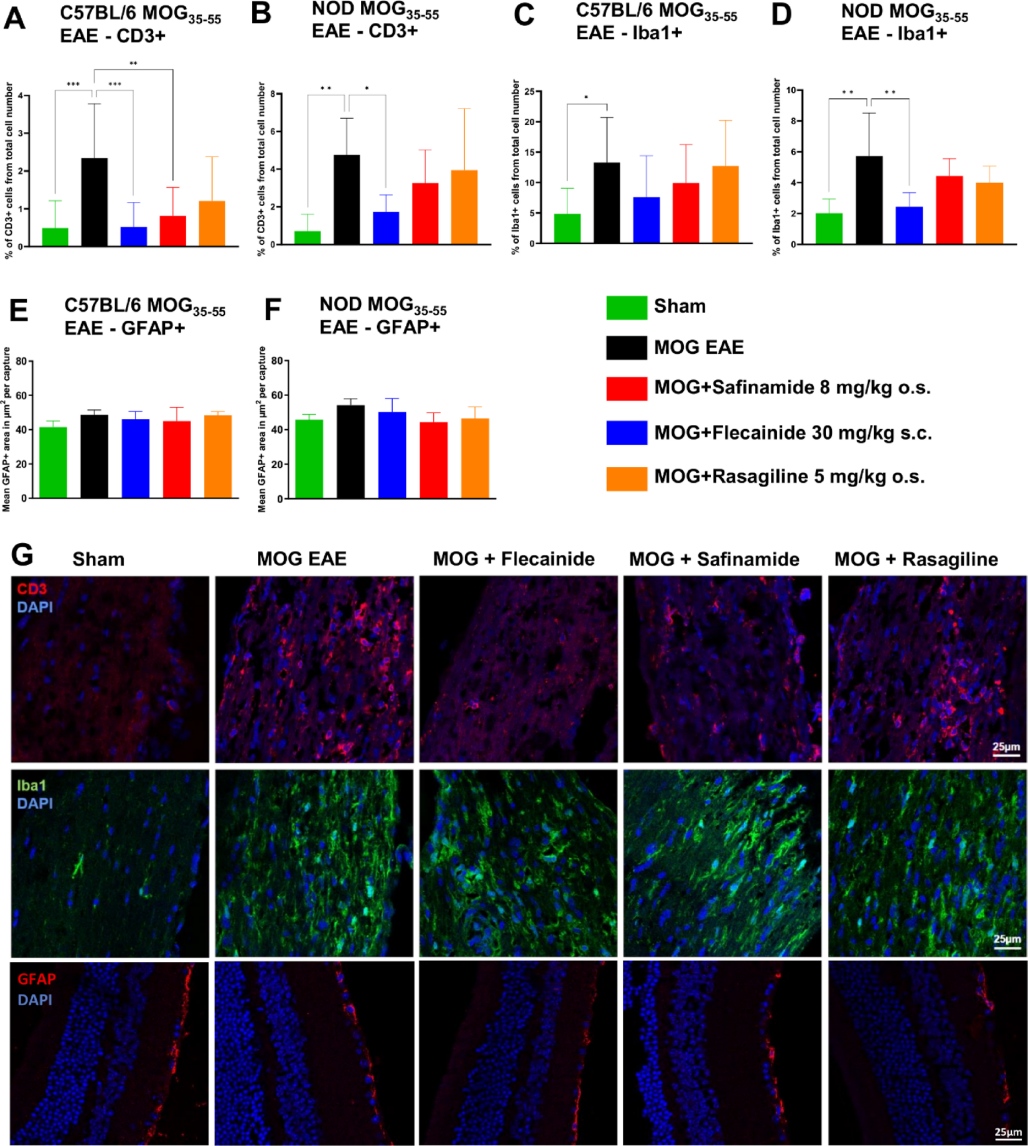
Histological evaluations of immune- and glial cell markers shows immune modulating effects of flecainide. Quantitative analyses (**A**–**F**) and representative illustrations (**G**) of staining for T-cell infiltration (CD3), and microglial activation (Iba1) from longitudinal sections of optic nerves in C57BL/6J and NOD mice, and staining for astrocytic activation (GFAP) in retinal cross sections in C57BL/6J mice, 90 days post-immunization. Representative images show sections from C57BL/6J mice. Total cell number was measured based on automated DAPI cell counting with ImageJ2/Fiji. CD3 and Iba1 positive cells were counted by blinded raters. *p < 0.05, **p < 0.01, ***p < 0.001, evaluated using ANOVA with Dunnett’s post hoc test compared to MOG EAE. n = 9 per group.

We extended our investigation to the histological evaluation of immune cell markers, specifically CD3, a marker for T-cell infiltration, and Iba1 (ionized calcium-binding adaptor molecule 1), indicative of microglial activation, in longitudinal sections of optic nerves from C57BL/6J and NOD mice 90 days post-immunization. In addition, GFAP (glial fibrillary acidic protein), a marker for astroglial activation, was analyzed in retinal cross sections. Quantitative analyses (A, B, C, D, E and F) and representative illustrations (G) are presented in Fig. 4. In C57BL/6J mice, treatment with both flecainide and safinamide significantly reduced the infiltration of CD3-positive lymphocytes compared to the MOG EAE control group, with flecainide achieving a MD of 1.8220 ± 0.4116 (*P* = 0.0005) and safinamide achieving an MD of 1.5323 ± 0.4317 (*P* = 0.0048). In contrast, in NOD mice, only flecainide treatment significantly reduced CD3-positive infiltrates (MD = 3.0239 ± 1.0146, *P* = 0.0228). No significant differences in Iba1 expression were observed among treatment groups in C57BL/6J mice. However, in NOD mice, flecainide treatment resulted in a significant reduction in Iba1-positive cells compared to the vehicle-treated EAE group (MD = 3.2844 ± 0.8723, *P* = 0.0036). No significant differences in GFAP expression were observed between treatment groups in either C57BL/6J or NOD mice, indicating that astrocytic activation remained unaffected by flecainide or safinamide treatment. These findings indicate that both flecainide and safinamide effectively reduce T-cell infiltration in the C57BL/6J EAE model, while flecainide additionally diminishes both T-cell infiltration and myeloid cell activation in the NOD-EAE model. This implies potential anti-inflammatory and neuroprotective effects of these treatments in the context of EAE.

### Histological analyses of neuronal and myelin markers in the optic nerve

**Fig. 5:**
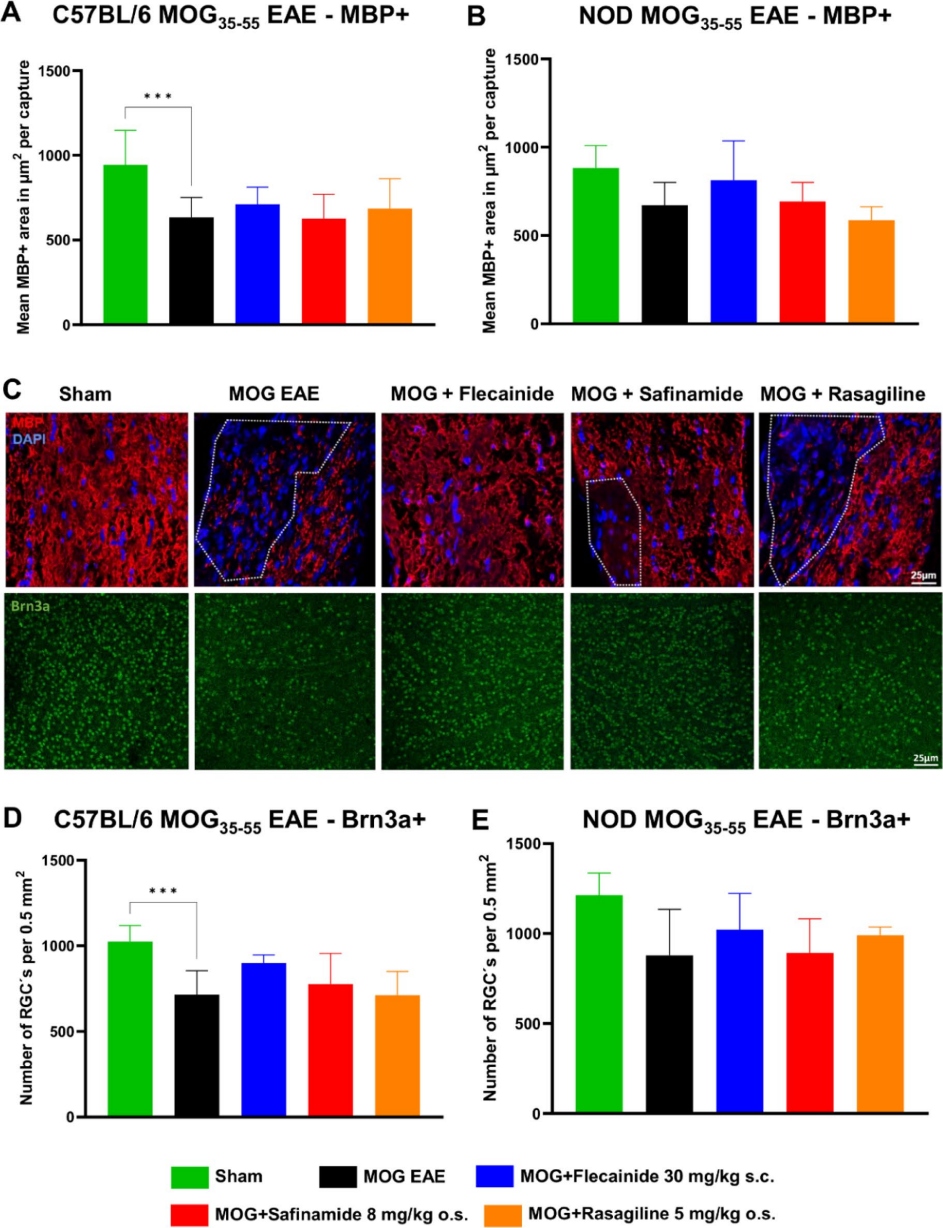
Histological evaluations of neuronal and myelin markers shows no difference after treatment. Quantitative analyses (**A**, **B**, **D**, **E**) and representative illustrations (**C**) of staining for myelination (MBP) and neuronal survival (RGC´s) (Brn3a) from longitudinal sections of optic nerves in C57BL/6J and NOD mice, 90 days post-immunization. Representative images show sections from C57BL/6J mice. The indicated µm^2^ mean of MBP + total area has been calculated out of a total 8.100 µm2 captured area. Brn3a positive cells were counted automated by ImageJ2 / Fiji. ***p < 0.001, evaluated using ANOVA with Dunnett’s post hoc test compared to MOG EAE. n = 9 per group.

In addition to assessing immune cell markers, we conducted histological evaluations of myelination and neuronal survival using Myelin Basic Protein (MBP) and Brain-specific homeobox/POU domain protein 3A (Brn3a) markers, respectively. Quantitative analyses (A, B, D, E) and representative illustrations (C) are presented in Fig. 5, based on longitudinal sections of optic nerves from C57BL/6J and NOD mice 90 days post-immunization. MBP, essential for nerve myelination, was utilized to evaluate myelin status, while Brn3a, expressed in retinal ganglion cells (RGCs), was employed to assess neuronal survival in the context of optic neuritis. Our results showed no significant differences in the levels of either MBP or Brn3a across treatment groups in both mouse models. However, there was a noticeable trend suggesting that flecainide may have beneficial effects on myelination and neuronal survival, as indicated by increased MBP and Brn3a expression, though these changes did not reach statistical significance.

### Assessment of CNS immune cell infiltration

**Fig. 6:**
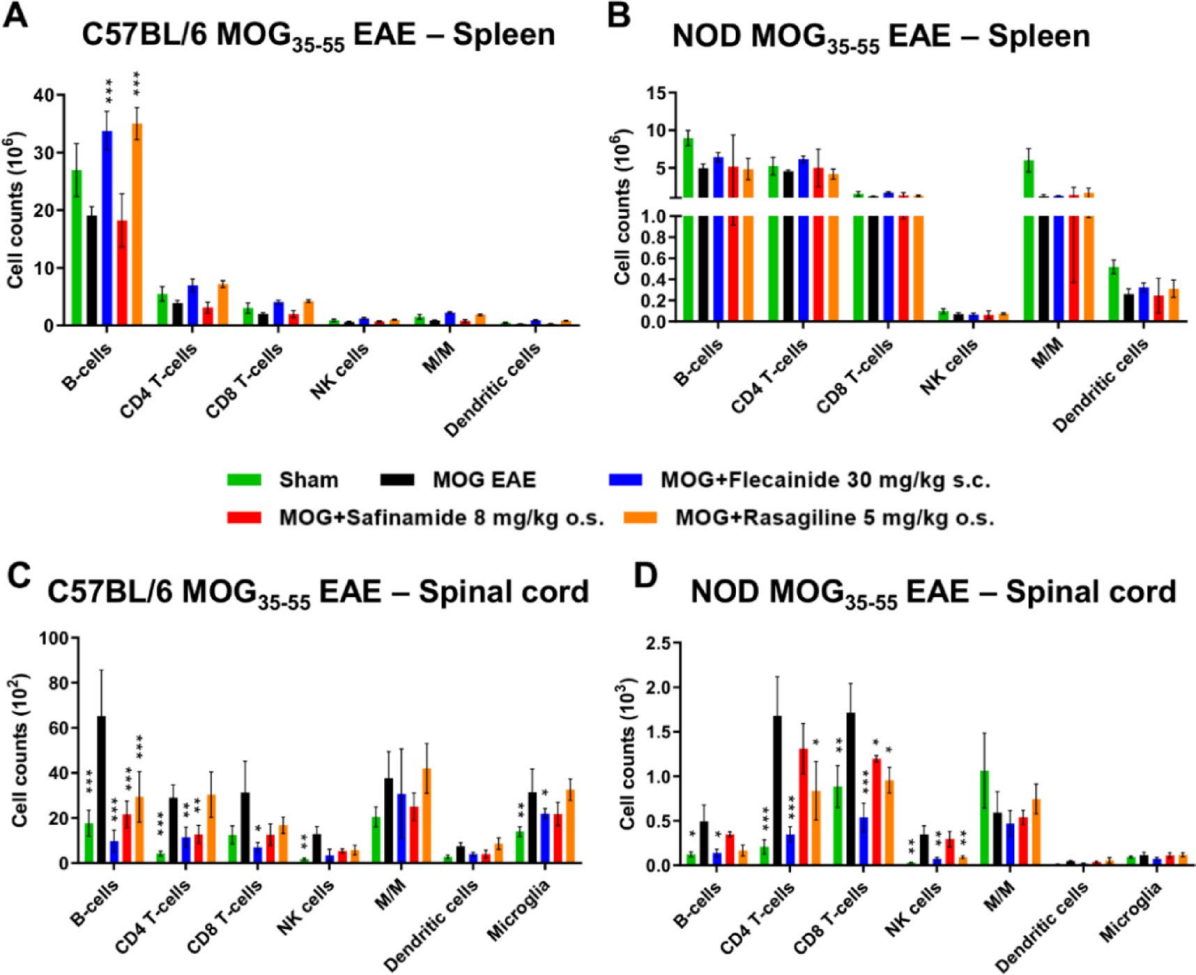
CNS immune cell infiltration analysis revealed BBB modulating effect of flecainide. Immunophenotyping using flow cytometry (CD45 + pre-gated) in C57BL/6J and NOD EAE mice 90 days post-immunization, focusing on spleen (**A**) and spinal cord (**B**). *p < 0.05; **p < 0.01; ***p < 0.001, as determined by ANOVA with Dunnett’s post hoc test in comparison to untreated MOG EAE mice. n = 9 per group. M/M = Monocytes/Macrophages.

Further investigation focused on analyzing immune cell infiltration across the BBB using immunophenotyping via flow cytometry (CD45 + pre-gated). We examined the spleen (Fig. 6A,B) and spinal cord (Fig. 6C,D) of C57BL/6J and NOD EAE mice 90 days post-immunization. In the spleen of C57BL/6J mice, treatment with flecainide (MD = 14.7039 ± 1.5572, *P* < 0.0001) and rasagiline (MD = 15.9590 ± 1.4776, *P* < 0.0001) resulted in a significant increase in B cells. Additionally, there was a trend towards increased counts of CD4+T cells, NK cells, microglia, and dendritic cells with flecainide treatment, although these changes were not statistically significant. In NOD mice, flecainide treatment showed a non-significant trend of overall immune cell increase in the spleen. In contrast, immune cell infiltration in the CNS, specifically the spinal cord, exhibited different dynamics. In C57BL/6J mice, all treatment groups demonstrated significant reductions in B cells (flecainide: MD = 55.5006 ± 9.5138, *P* < 0.0001; safinamide: MD = 43.6541 ± 9.0240, *P* < 0.0001; rasagiline: MD = 35.8577 ± 11.3321, *P* = 0.0005). Additionally, safinamide (MD = 16.2063 ± 9.0249, *P* = 0.0020) and flecainide (MD = 17.5004 ± 9.5138, *P* = 0.0093) significantly reduced CD4+T cells, while flecainide also significantly decreased CD8 + T cells (MD = 24.2563 ± 3.2195, *P* = 0.0193) and microglia (MD = 9.5011 ± 1.4139, *P* = 0.0215). In NOD mice, flecainide led to significant reductions in B cells (MD = 0.3559 ± 0.0549, *P* = 0.0395), CD4 + T cells (MD = 1.3310 ± 0.0916, *P* = 0.0007), CD8+T cells (MD = 1.1782 ± 0.1873, *P* = 0.0004), and NK cells (MD = 0.2775 ± 0.0230, *P* = 0.0049). Safinamide significantly reduced CD8+T cells (MD = 0.5181 ± 0.0294, *P* = 0.0394), while rasagiline treatment significantly reduced CD4+T cells (MD = 0.8432 ± 0.3520, *P* = 0.0452), CD8+T cells (MD = 0.7590 ± 0.1819, *P* = 0.0207), and NK cells (MD = 0.2569 ± 0.0036, *P* = 0.0069). These findings indicate that the treatment compounds, particularly flecainide, resulted in increased numbers of immune cells in the spleen and reduced numbers in the spinal cord.

### Comprehensive analysis of BBB modulation through in vitro gene expression and permeability assay in pMBMECs and in vivo Evan’s Blue Dye assay

**Fig. 7:**
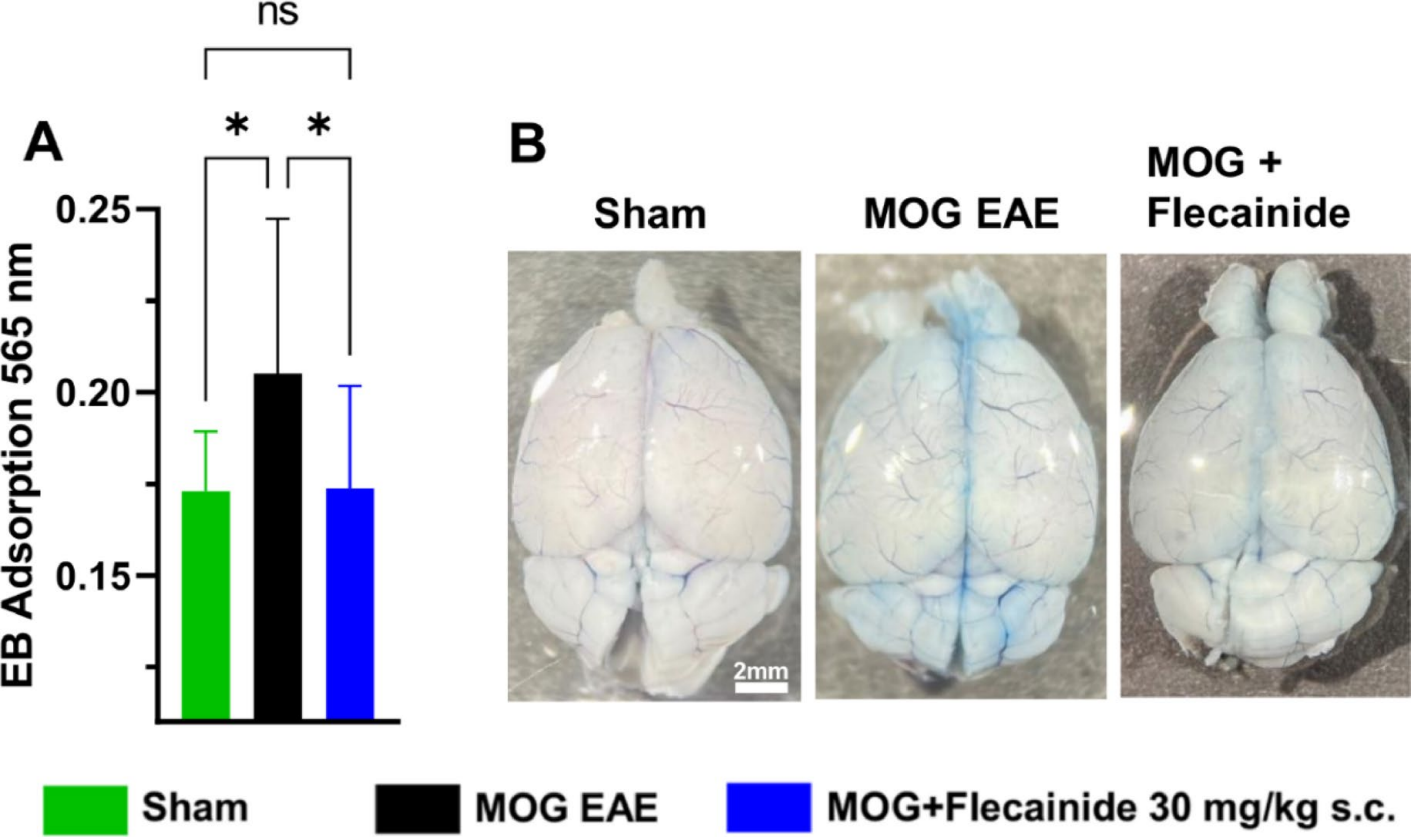
Analysis of Blood–Brain Barrier permeability using Evan’s Blue Assay after EAE Induction shows beneficial effects of flecainide. Quantitative data (**A**) and representative images (**B**) depict Evan’s Blue (EB) absorption at 565 nm from mice whole brains homogenate after weight correction, sacrificed at day 18 post-EAE induction at the peak of disease. Statistical significance is represented by *p < 0.05 and ns (not significant), determined by ANOVA followed by Dunnett’s post hoc test comparing to untreated MOG EAE mice. N = 15 per group. Scale bar: 2mm.

Based on flow cytometry results showing reduced lymphocyte infiltration into the CNS after flecainide treatment, we assessed the integrity of the BBB using the EB-assay (Fig. 7). Quantification revealed a significant increase in EB perfusion in the CNS of MOG EAE mice. In contrast, flecainide treatment led to a noticeable reduction in EB perfusion (MD = 0.0315 ± 0.0113, *P* = 0.0167) compared to untreated MOG EAE animals.

**Fig. 8:**
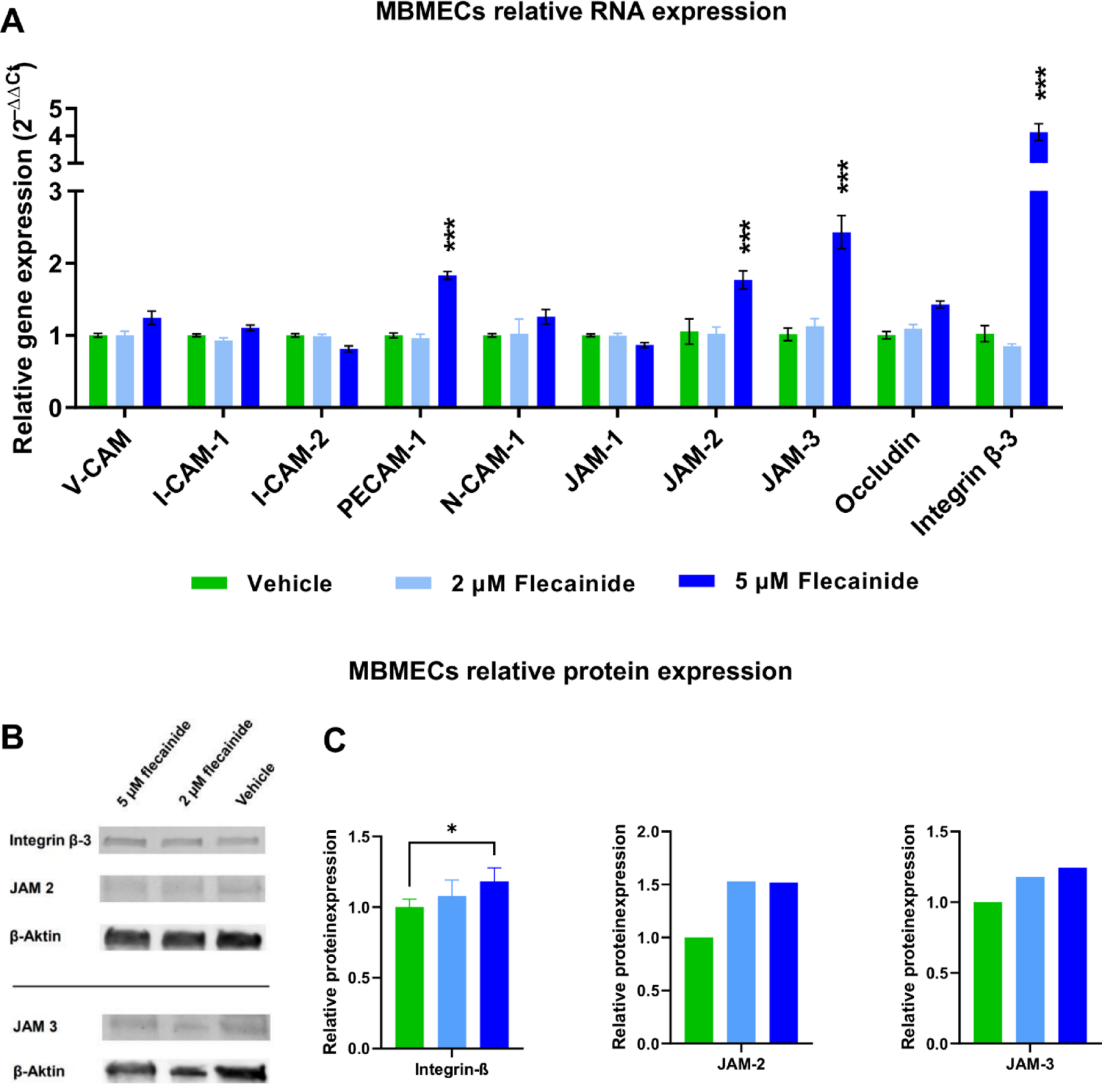
In vitro flecainide treatment of pMBMECs alters gene expression profile and protein expression of important BBB integrity markers. (**A**) The graph depicts the ΔΔCt values (normalized to housekeeping gene GAPDH and relative to vehicle-treated controls) for a range of genes of interest (X-axis) in pMBMECs. The treatment groups include cells exposed to 2 µM or 5 µM flecainide or PBS vehicle control 24 h prior to cell harvesting. The bars represent the mean ΔΔCt values while error bars indicate standard error of the mean (SEM) across three independent experiments. (**B**) Representative Western blot images showing protein expression of integrin β-3, JAM2, and JAM3 in pMBMECs treated with flecainide (2 µM or 5 µM) or vehicle. (**C**) Densitometric quantification of protein expression normalized to β-actin and additionally to the vehicle-treated control group. Statistical significance was determined using two-way ANOVA followed by Bonferroni´s post-hoc test. Asterisks denote significant differences compared to the vehicle-treated group at *p < 0.05 and ***p < 0.001.

To evaluate the impact of flecainide on gene expression in pMBMECs, we examined the RNA expression of key genes depicted in Fig. 8. These genes include *v-cam*, which facilitates leukocyte adhesion; *i-cam-1* and *i-cam-2,* involved in leukocyte trafficking; *pecam-1* (CD31), essential for angiogenesis and BBB integrity; *n-cam-1*, important for neuronal development; *jam-1*, *jam-2* and *jam-3*, critical for tight junctions and barrier function; *occludin*, a tight junction component in endothelial cells; and *integrin β-3*, key in cell adhesion and signal transduction^24–26^. In cells treated with 2 µM flecainide, no significant differences were observed in the relative expression of these genes compared to vehicle-treated controls. However, cells treated with 5 µM flecainide exhibited significant increases in the expression of several genes. *Pecam-1* showed a substantial increase with a MD of 0.8305 ± 0.0059 (*P* = 0.0004), and *jam-2* expression was elevated with an MD of 0.7141 ± 0.0168 (*P* = 0.0003). Additionally, *jam-3* expression significantly increased with an MD of 1.416 ± 0.0401 (*P* = 0.0002), and *integrin β-3* demonstrated the most substantial increase with an MD of 3.111 ± 0.0348 (*P* = 0.0001).

To validate the transcriptional changes observed in the qPCR analysis, a subsequent Western blot was performed targeting selected molecules that had shown differential expression following flecainide treatment. Specifically, protein levels of JAM-2, JAM-3, and integrin β-3 were assessed, as these genes were significantly upregulated at the mRNA level upon exposure to 5 µM flecainide. The results confirmed that JAM-2 and JAM-3 exhibited trends toward increased expression at the protein level, while integrin β-3 showed a significant upregulation in the 5 µM flecainide group compared to vehicle-treated controls (MD =  − 0.1816 ± 0.0607, *P* = 0.0362), consistent with the qPCR findings. PECAM-1 protein could not be reliably detected under the given experimental conditions. For JAM-2 and JAM-3, protein lysates from two mice were pooled per well, resulting in one biological replicate per group. For integrin β-3, eight mice per group were used, pooled into four wells (two mice per well), providing a more robust dataset. These findings support the notion that flecainide modulates adhesion molecule expression in pMBMECs, not only at the transcript level, but also at the protein level (Fig. 8B,C).

**Fig. 9:**
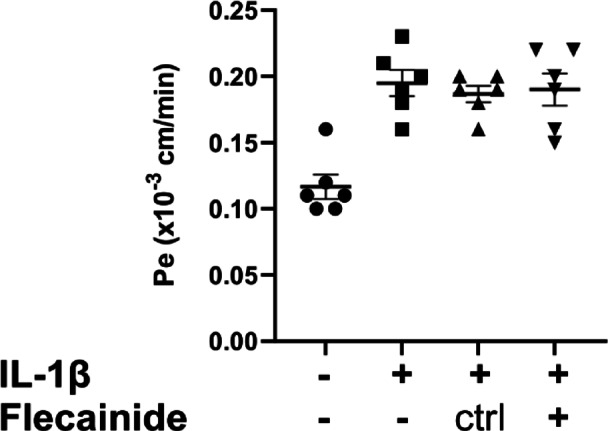
Flecainide treatment does not change the permeability of IL-1β stimulated pMBMECs. The graph shows the permeability coefficient (Pe) value of pMBMECs to 3 kDa Dextran. Where indicated, IL-1β stimulation was at 20 ng/ml for 16 h, and Flecainide treatment was at 5 µM for 24 h. Ctrl, vehicle control. For each condition, 6 data points deriving from 2 independent experiments are shown. Error bar shows ± SEM. No significant differences were found between Flecainide and vehicle-treated groups using ordinary one-way ANOVA.

We performed permeability experiments to investigate the effect of flecainide on BBB permeability under inflammatory conditions, modeled in vitro using IL-1β-stimulated pMBMECs (Fig. 9). IL-1β treatment significantly compromised the barrier properties of pMBMECs (MD = 0.07833, *P* < 0.0001)^27^. However, flecainide treatment did not significantly alter the permeability of IL-1β-stimulated pMBMECs compared to the DMSO vehicle (MD = 0.003333 ± 0.037925, *P* = 0.9946) or IL-1β treatment alone (Fig. 9).

**Fig. 10:**
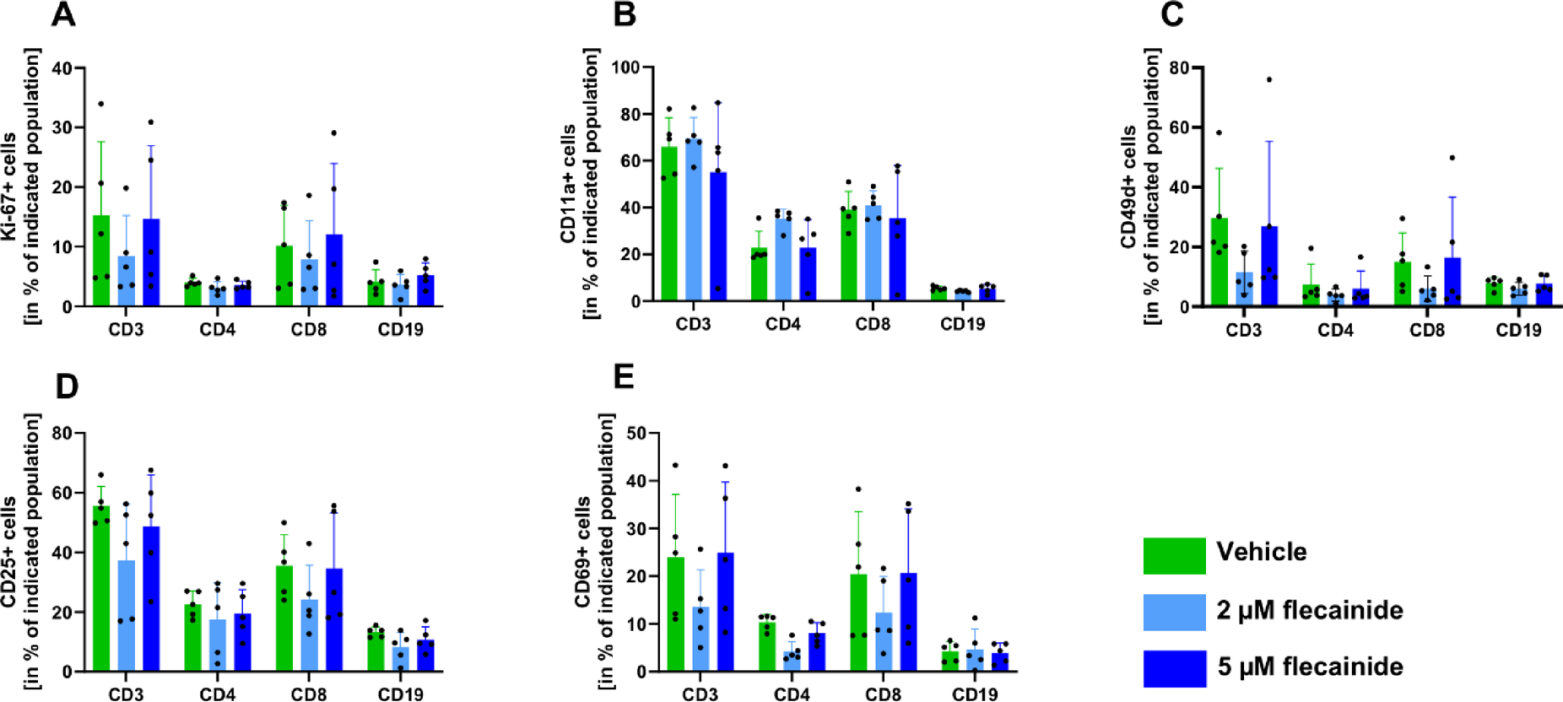
Flow cytometric analysis of lymphocyte activation, adhesion, and proliferation markers after flecainide treatment. Splenocytes from mice treated with vehicle, 2 µM, or 5 µM flecainide were stimulated with CD3/CD28 antibodies and analyzed for expression of the proliferation marker Ki-67 (**A**), adhesion molecules CD11a (**B**) and CD49d (**C**), and activation markers CD25 (**D**) and CD69 (**E**) within CD3⁺, CD4⁺, CD8⁺, and CD19⁺ cell populations. No significant differences were observed between treatment groups. Bars represent mean ± SD. n = 5 mice per group.

Figure 10 illustrates flow cytometry analyses of proliferation marker (Ki-67), adhesion molecules (CD11a, CD49d), and activation markers (CD25, CD69) in CD3+, CD4+, CD8+, and CD19+splenocytes. No significant differences were observed in the expression of these markers following treatment with either 2 µM or 5 µM flecainide compared to vehicle-treated controls. These findings suggest that flecainide does not exert measurable immunomodulatory effects on the activation, proliferation, or adhesion molecule expression of T or B lymphocytes in the spleen. This further supports the hypothesis that the observed therapeutic effects of flecainide are not mediated via direct action on peripheral lymphocytes.

**Fig. 11:**
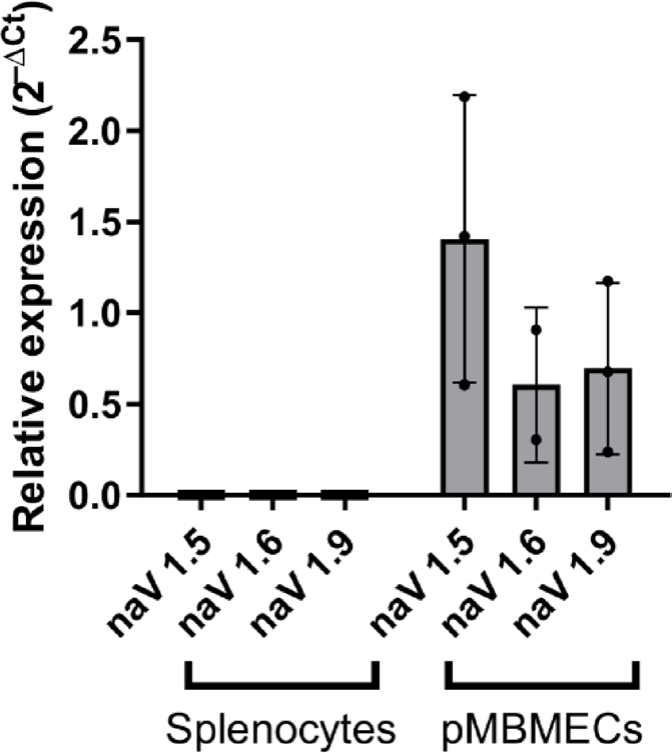
Relative expression of sodium channel isoforms in splenocytes and pMBMECs. Relative expression of sodium channel isoforms in splenocytes and pMBMECs. qPCR analysis of NaV1.5, NaV1.6, and NaV1.9 expression in splenocytes and pMBMECs. While no expression of any tested NaV isoform was detected in splenocytes, all three channels were expressed in pMBMECs, with NaV1.5 showing the highest relative levels. Data are presented as 2^–ΔCt^ values normalized to GAPDH. Bars represent mean ± SD from n = 3.

This lack of immunomodulatory activity on splenocytes is consistent with the qPCR findings shown in Fig. 11, where expression levels of voltage-gated sodium channels NaV1.5, NaV1.6, and NaV1.9 were assessed in splenocytes and pMBMECs. Notably, none of the analyzed NaV channels were detectably expressed in splenocytes, while all three showed marked expression in pMBMECs (Mean = 0.9008; SD = 0.4384). These data provide a molecular rationale for the cell type-specific action of flecainide, reinforcing the interpretation that its primary target in this context is the endothelial compartment rather than lymphocytes.

## Discussion

EAE is a widely recognized and extensively validated animal model of MS, characterized predominantly by neuroinflammation, demyelination, and neurodegeneration^1^. However, it is important to acknowledge that EAE only partially recapitulates the complex immunopathology and chronic disease course observed in human MS. The translational relevance of preclinical findings must therefore be interpreted with caution, particularly in light of previous therapeutic approaches that showed efficacy in EAE but failed in clinical trials. For example, sodium channel blockers such as lamotrigine^28^ and acid-sensing ion channel inhibitors like amiloride^28^ demonstrated promising neuroprotective effects in animal models but did not meet endpoints in progressive MS patients. Our study does not include experiments on human brain tissue or human endothelial cell lines, and we refrain from making direct claims about clinical efficacy in MS. Instead, we view our findings as a preclinical proof of concept demonstrating BBB-stabilizing and neuroprotective properties of flecainide in the context of CNS inflammation, which warrant further validation in human-based systems. Notably, there are examples of NaV blockers where translational success has been achieved. Phenytoin, another sodium channel blocker that was also assessed and found effective in our study, demonstrated significant neuroprotective effects in a Phase 2 clinical trial in patients with acute optic neuritis, a common manifestation of autoimmune CNS inflammation^29^. This suggests that, at least in specific MS-related contexts such as optic neuritis, findings from EAE models can indeed translate into meaningful clinical benefit.

In this study, we systematically investigated the therapeutic potential of MAO-B inhibition and sodium channel blockade, both as individual interventions and in combination, across multiple murine models of EAE. Specifically, our research focused on flecainide, a Class Ic antiarrhythmic agent primarily utilized clinically for managing ventricular arrhythmias due to its potent NaV 1.5 channel blocking properties. Prior preclinical studies have demonstrated that both safinamide and flecainide exhibit beneficial effects in EAE, thus highlighting their potential therapeutic relevance for MS^10,13^.

NaV channels are prominently expressed in electrically excitable cells, including neurons, skeletal muscle fibers, and cardiac myocytes, where they play a fundamental role in action potential initiation and propagation. However, emerging evidence indicates their expression at significantly lower densities in various non-excitable cell populations, suggesting broader physiological roles beyond classical electrophysiological functions^30^. Despite extensive investigation, the precise mechanistic pathways underlying the therapeutic efficacy of sodium channel blockers such as flecainide and safinamide in EAE remain incompletely understood. In particular, uncertainty persists regarding whether their beneficial effects primarily result from direct actions on immune cells, modulation of neuronal excitability, or through influence on other cell types critically involved in disease progression, notably those contributing to the integrity of the BBB. BBB disruption represents a central pathological feature of EAE, critically influencing disease progression by facilitating inflammatory cell infiltration into the CNS. Hence, clarifying the precise cellular and molecular targets mediating the protective effects of sodium channel blockade in EAE is essential for the rational development of targeted therapeutic strategies in neuroinflammatory diseases.

Phenytoin, a Class Ib sodium channel blocker, has previously demonstrated notable beneficial effects in EAE models. Evidence from preclinical studies suggests that phenytoin effectively protects CNS axons, positioning it as a promising candidate for treating neuroinflammatory conditions^29,31^. The before mentioned^29^ clinical trial investigating phenytoin in patients with acute optic neuritis yielded positive outcomes. However, our data revealed a superior therapeutic effect of flecainide over phenytoin, as shown by significantly enhanced disease mitigation and functional recovery parameters. Given these results, we choose to continue our subsequent investigations focusing on flecainide, the Class Ic sodium channel blocker, due to its demonstrated greater efficacy in alleviating EAE-associated pathology and symptoms. In this study, we introduce a novel hypothesis proposing a direct modulatory effect of sodium channel blockers on BBB integrity. Our experimental data support the conclusion that flecainide enhances BBB stability, thereby significantly reducing lymphocyte infiltration into the CNS. Furthermore, the observed protective effects of safinamide in our EAE models appear primarily mediated through sodium channel blockade rather than MAO-B inhibition, as evidenced by the lack of efficacy observed with the selective MAO-B inhibitor rasagiline. Notably, both flecainide (with substantial effects) and safinamide (with comparatively moderate effects) demonstrated significant improvements in disability progression, neurodegeneration, visual function, immune cell infiltration, and BBB integrity.

These in vivo outcomes, particularly the pronounced effects on visual function and retinal neurodegeneration, have important clinical implications. Firstly, our results indicate that flecainide and safinamide may serve as viable candidates for clinical trials targeting acute optic neuritis, employing OCT and visual function assessments as primary endpoints. Secondly, as prior evidence has shown correlations between therapeutic outcomes in optic neuritis and effects in other CNS regions, such as the spinal cord^7,21,32,33^, our data may imply broader neuroprotective benefits that extend to general disability mitigation in EAE. Finally, the capacity of these agents to reduce long-term disability resulting from acute inflammatory episodes is particularly significant, given that current therapeutic strategies for inflammatory CNS diseases primarily target acute attacks without effectively preventing subsequent neurodegeneration. Despite these promising results, it remains critical to acknowledge that EAE serves only as an animal model for autoimmune CNS disorders and does not replicate every aspect of MS pathology. However, the demonstrated clinical efficacy of sodium channel blockade in human trials of acute optic neuritis with phenytoin lends strong support to the potential translatability of our findings. Our data further suggest that flecainide may represent a superior therapeutic alternative to phenytoin, thereby warranting detailed exploration in future clinical trials.

In terms of immune response, our histological analyses revealed that both flecainide and safinamide significantly reduced T-cell infiltration in the optic nerves of C57BL/6J EAE mice. T-cell infiltration is known to contribute substantially to the pathogenic mechanisms driving neuroinflammation in neuropathologic conditions such as EAE. Additionally, flecainide treatment also resulted in a significant reduction of both T-cell infiltration and microglial activation in the NOD-EAE model. These outcomes are consistent with previous reports highlighting the ability of safinamide and flecainide to mitigate microglial activation in other preclinical MS models^13^. Notably, our study further identified that both treatments, with flecainide showing a more pronounced effect, led to increased immune cell populations in the spleen concomitant with decreased immune cell presence in the spinal cord. This suggests that sodium channel blockers potentially modulate immune cell distribution, reducing infiltration across the BBB. This immunological dichotomy aligns with gene expression analyses performed on pMBMECs following flecainide exposure. Specifically, treatment with 5 µM flecainide significantly upregulated genes known to be crucial for maintaining BBB integrity, further supporting the hypothesis of barrier modulation under neurodegenerative conditions. Interestingly, despite these transcriptional alterations, permeability assay using IL-1β-stimulated pMBMECs did not reveal immediate functional improvements in barrier integrity. This discrepancy suggests that in vivo conditions likely involve additional cellular or structural elements influencing BBB function, including interactions among endothelial cells, astrocytes, microglia, neurons, and inflammatory mediators^34^. Furthermore, temporal aspects must be considered, as gene expression changes may require extended periods to manifest fully as measurable functional outcomes. It should also be noted that while IL-1β is widely used to model inflammation-induced BBB dysfunction in vitro^35,36^, it represents only a subset of the pro-inflammatory stimuli active in EAE. In particular, T cells and their secreted cytokines are likely to contribute significantly to the endothelial response in vivo. Therefore, the lack of flecainide effects under IL-1β conditions may reflect the limited scope of the model. Moreover, the absence of glial and immune cell interactions in such monocellular systems further constrains their ability to detect complex, indirect mechanisms—such as the glia-mediated BBB stabilization. This interpretation is supported by our in vivo assessments of BBB permeability using Evans Blue dye, which revealed reduced vascular leakage following flecainide treatment in the MOG-EAE model. While we acknowledge that Evans Blue provides a coarse and unspecific measure of global endothelial permeability—and does not allow conclusions regarding the precise anatomical sites of immune cell transmigration—the observed reduction in dye extravasation is consistent with a more intact vascular barrier. Importantly, this decrease in leakage correlated with diminished immune cell infiltration as shown by flow cytometry, supporting the notion of overall improved barrier function. These findings underline the complexity of BBB regulation in vivo and emphasize the necessity of physiologically integrated models when evaluating therapeutic interventions in neuroinflammation. No significant differences were observed in myelination and neuronal survival analyzed histologically across the treatment groups. While there were trends towards benefits from flecainide treatment, this aspect of our findings warrants further investigation.

In previous studies, significant efforts have been made to understanding the mechanisms underlying the beneficial effects of sodium channel blockers in neuroinflammatory conditions. Bechtold et al.^10^ initially proposed a critical relationship between the extent of neuroinflammation and subsequent axonal degeneration, suggesting that inflammatory mediators may induce an intra-axonal accumulation of sodium and calcium ions, thereby triggering axonal injury. This hypothesis provided an important conceptual foundation for the exploration of sodium channel blockers as therapeutic agents aimed at mitigating neurodegeneration. Furthermore, Craner et al.^12^ elaborated on this idea by emphasizing the significant role played by activated microglia and macrophages in neuroinflammatory processes. Their work suggested that sodium influx via voltage-gated sodium channels could facilitate increased intracellular calcium concentrations within microglia, consequently enhancing their inflammatory responses and exacerbating neuronal injury. Building upon these insights, the research by Morsali et al.^13^ demonstrated that safinamide and flecainide, offer substantial neuroprotective benefits through their dual actions on reducing microglial activation and protecting axonal integrity. Although the precise molecular mechanisms underlying these protective effects remain incompletely characterized, their findings clearly show the potential clinical value of sodium channel blockade in preserving white matter integrity in neuroinflammatory contexts.

Our present findings expand on these earlier insights, providing compelling evidence that sodium channel blockers may also exert significant therapeutic effects through the modulation of BBB integrity. We suggest that BBB stabilization represents an important additional pathway, complementing the known axonal and immunomodulatory mechanisms. Our data strongly support the idea that the beneficial effects observed with flecainide and, to a lesser extent, safinamide, in EAE models can be attributed, at least in part, to their influence on BBB integrity. Thus, sodium channel blockers emerge as particularly attractive therapeutic candidates due to their multifaceted mode of action, encompassing not only direct neuronal and immunological effects but also the novel, substantial modulation of the BBB. This integrated mechanistic view reinforces the potential of sodium channel blockers as promising therapeutic strategies in the clinical management of neuroinflammatory disorders.

**Fig. 12:**
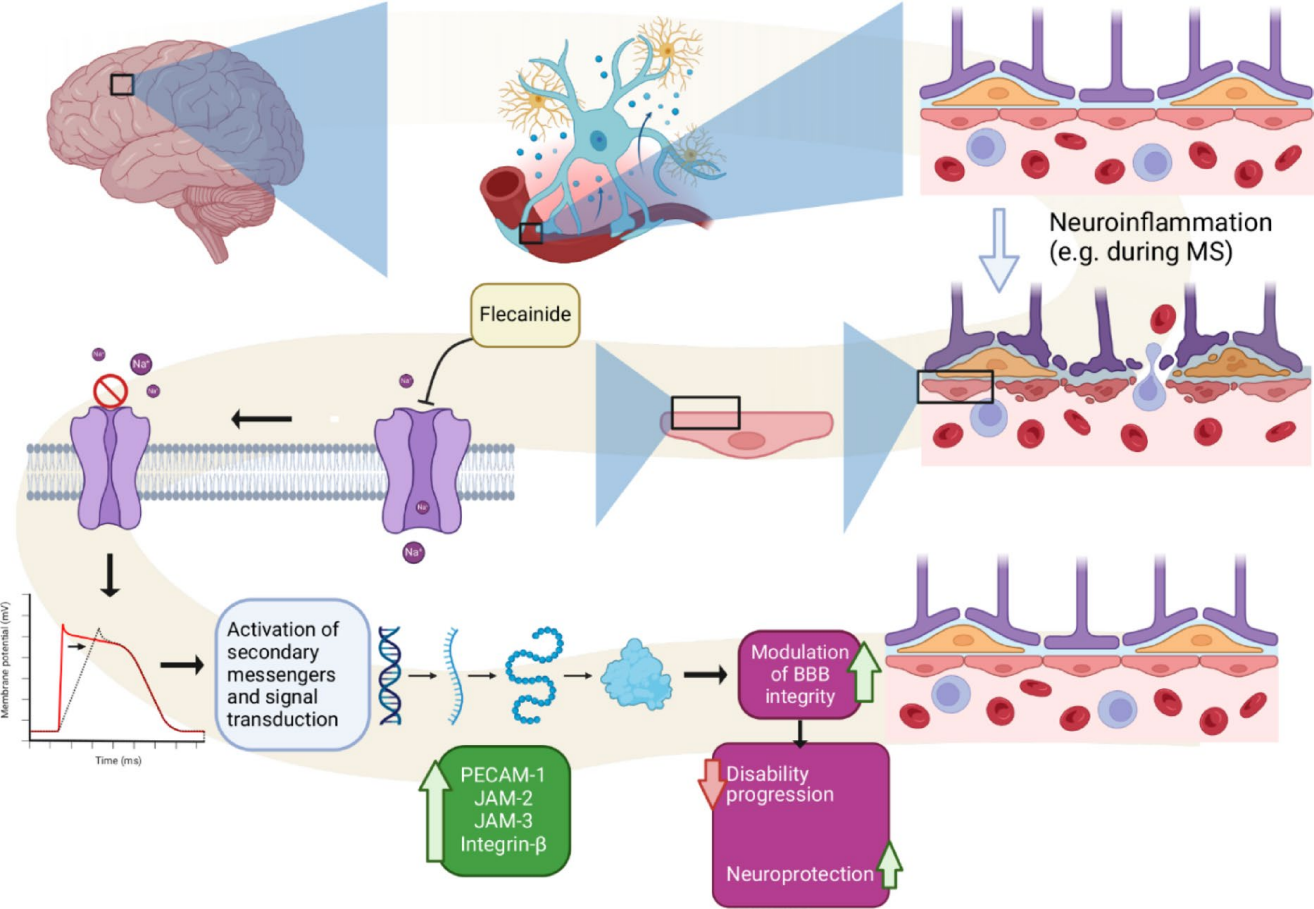
Proposed mechanism of action of flecainide under neuroinflammatory conditions: Flecainide, a Class Ic antiarrhythmic agent, confers neuroprotection in the context of neuroinflammation by selectively inhibiting endothelial NaV 1.5. This targeted blockade leads to upregulation of junctional and adhesion molecules, thereby enhancing BBB integrity. As a result, immune cell infiltration into the central nervous system is reduced, attenuating neuroinflammation and contributing to improved functional outcomes.

Flecainide, phenytoin, and safinamide each demonstrate considerable therapeutic potential in the context of neuroinflammatory conditions, by effectively mitigating disease progression and exerting neuroprotective effects by modulating immune responses in the CNS through maintaining the integrity of the BBB, as summarized in Fig. 12. Previous investigations suggest that the neuroprotective properties of these sodium channel blockers may involve mechanisms such as enhanced antioxidant defenses, decreased microglial superoxide production, and elevated glutathione levels, although the complete molecular pathways responsible for these effects are not clarified^13^.

To investigate the cellular target of flecainide, we performed a qPCR-based screen for various isoforms of NaVs in splenocytes. In line with previous reports^37^, none of the analyzed isoforms were detectably expressed in these immune cells, suggesting a lack of functionally relevant sodium channel activity. Consistent with this molecular profile, flecainide treatment did not alter splenocyte proliferation, activation status, or adhesion molecule expression, arguing against a direct immunomodulatory effect on peripheral immune cells via NaV1.5. In contrast, robust expression of NaV1.5, NaV1.6, and NaV1.9 was observed in pMBMECs. Given the pivotal role of brain endothelial cells in maintaining BBB integrity, these data support the hypothesis that flecainide exerts its effects primarily through modulation of endothelial function. This interpretation is further supported by Western blot analyses demonstrating elevated protein levels of several adhesion- and junction-associated molecules—specifically integrin-β-3, JAM-2, and JAM-3—following flecainide treatment. Recent studies highlight a central role of glial cells in regulating BBB function during neuroinflammation^38,39^. Performed GFAP staining of optic nerve tissue of EAE mice revealed no differences between flecainide- and vehicle-treated animals, indicating no effect on astrocytic activation. In contrast, Iba1 staining showed reduced microglial activation in the flecainide group, which likely reflects an indirect consequence of diminished immune cell infiltration into the CNS rather than direct modulation of microglia. Taken together, these findings support the conclusion that pMBMECs, rather than immune or glial cells, represent the primary target of flecainide, mediating its neuroprotective effects by stabilizing the BBB and limiting CNS inflammation.

Our findings highlight flecainide, a potent sodium channel 1.5 blocker, as particularly promising due to its pronounced modulatory effects on BBB integrity, in addition to its known effects on axonal protection and immune cell regulation. Collectively, these multi-dimensional therapeutic actions position NaV blockers as attractive candidates for a comprehensive treatment strategy in neuroinflammatory conditions. This multifaceted therapeutic profile not only aligns with and extends existing conceptual frameworks but also broadens the therapeutic scope by addressing multiple pathological targets simultaneously. Therefore, further research is essential to clarify detailed mechanistic pathways, optimize treatment strategies, and fully leverage the therapeutic potential of NaV blockers in the clinical management of neuroinflammatory disorders.

## Supplementary Information

Below is the link to the electronic supplementary material.

Supplementary Material 1

## Data Availability

All data from this study can be made available upon request to qualified researchers by contacting Mustafa Sindi at must.sindi@gmail.com.

## Abbreviations

ACK: Ammonium-chlorid-kalium (lysis buffer)
Brn3a: Brain-specific homeobox/POU domain protein 3A
BW: Body weight
CC: Cell count
CFA: Complete Freund’s adjuvants
CNS: Central nervous system
EBD: Evan’s Blue Dye
EAE: Experimental autoimmune encephalomyelitis
EDSS: Expanded disability status scale
ELM: External limiting membrane
FCS: Fetal calf serum
GAPDH: Glyceraldehyde 3-phosphate dehydrogenase (housekeeping gene)
GCL: Ganglion cell layer
GEE: Generalized estimation equation
GFAP: Glial fibrillary acidic protein
IBA1: Ionized calcium-binding adaptor molecule 1
I-CAM-1 and I-CAM-2: Intercellular adhesion molecules 1 and 2
IL-1β: Interleukin-1 beta
INL: Inner nuclear layer
IPL: Inner plexiform layer
IRL: Inner retinal layer
IS: Inner segment
JAM-1, JAM-2, and JAM-3: Junctional adhesion molecules -1, -2 and -3
LS: Large separation (column type in MACS)
MAO: Monoamine oxidase
MACS: Magnetic-activated cell sorting
MBP: Myelin basic protein
MD: Mean difference
MOG: Myelin oligodendrocyte glycoprotein
MOG35-55: Myelin oligodendrocyte glycoprotein fragment 35–55
MS: Multiple sclerosis
MT: Mycobacterium tuberculosis
NaV1.5/NaV1.6/NaV1.9: Voltage-gated sodium channel isoforms
N-CAM-1: Neural cell adhesion molecule 1
NFL: Nerve fiber layer
NOD: Non-obese diabetic
OCT: Optical coherence tomography
OMR: Optomotor response
ON: Optic neuritis
ONL: Outer nuclear layer
OPL: Outer plexiform layer
ORL: Outer retinal layer
OS: Outer segment
P: Probability value
pMBMECs: Primary mouse brain microvascular endothelial cells,
PECAM-1: Platelet endothelial cell adhesion molecule
PTX: Pertussis toxin
RPE: Retinal pigment epithelium
SEM: Standard error of the mean
TRT: Total retinal thickness
V-CAM: Vascular cell adhesion molecule 1
