# Supplementary material for: Flecainide mediated sodium channel blockade enhances blood brain barrier integrity and promotes neuroprotection in neuroinflammation
Source: Sci Rep. 2025 Aug 23;15:31032. doi: 10.1038/s41598-025-15430-w (PMC12375043; doi:10.1038/s41598-025-15430-w)
Supplement: Supplementary file 1 — Supplementary Material 1 [file 41598_2025_15430_MOESM1_ESM.pdf]

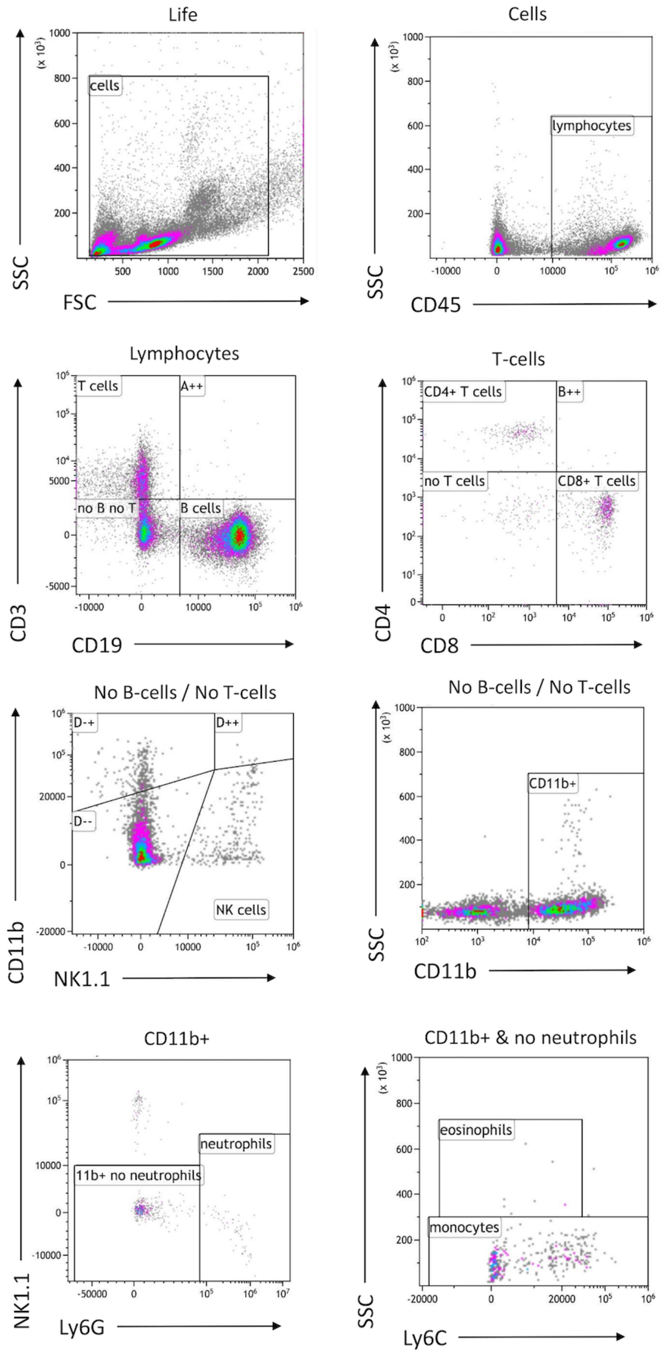

Figure S1: Flow cytometry: Gating strategy. Living cells were first identified based on side scatter (SSC) and forward scatter (FSC) properties. Subsequent gates identified cells based on CD45 expression (SSC vs. CD45). From the CD45+ population, lymphocytes were distinguished by gating CD3 vs. CD19. CD3+ T-cells were further identified within the lymphocyte population based on CD4 and CD8 expression (CD4 vs. CD8). For non-B and non-T cells, we used a gating strategy with CD11b vs. NK1.1 followed by SSC vs. CD11b. CD11b+ cells were further differentiated based on NK1.1 and Ly6G (NK1.1 vs. Ly6G) and SSC vs. Ly6C, allowing identification of monocytes.
